# A Mild and Modular Approach to the Total Synthesis of Desferrioxamine B

**DOI:** 10.1021/acs.joc.3c02739

**Published:** 2024-03-12

**Authors:** Todd E. Markham, Rachel Codd

**Affiliations:** School of Medical Sciences, The University of Sydney, Sydney, New South Wales 2006, Australia

## Abstract

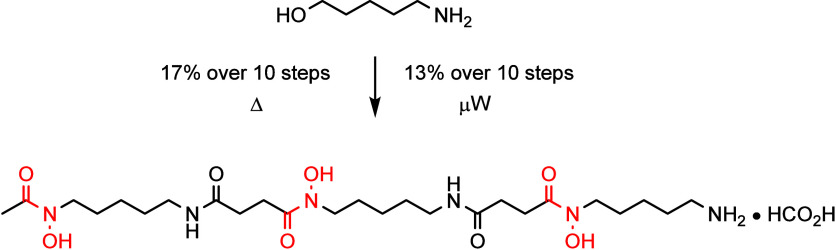

A mild and modular approach to the total synthesis of the WHO-listed essential medicine desferrioxamine B is described. Hydroxamic acid fragments were installed under mild conditions, a generalized divergent acylation procedure used to access two monomer precursors, and a transfer hydrogenation reaction used to unmask the hydroxamic acid moieties. Desferrioxamine B was generated over ten linear steps as the formate salt in 17% overall yield using standard amide coupling conditions or in 13% overall yield using microwave-assisted amide coupling conditions.

Siderophores make up a class of secondary metabolite produced by bacterial and fungal species.^1−3^ These metabolites serve to sequester iron from the environment, which is typically insoluble and not readily bioavailable, by forming soluble Fe^3+^ complexes that can then be imported into the cell.^4^ This class of natural product has broad chemical diversity; however, desferrioxamine B (DFOB, **1**) has particular notoriety due to its long-standing application in medicine and chemical biology.^5^

**1** is a small molecule that contains three hydroxamic acid units which are critical for its ability to coordinate Fe^3+^ (Figure 1).^6^ It was initially isolated in 1958 from *Streptomyces pilosus*(7) and has since been used as a therapeutic agent in the treatment of iron overload disease states.^5^ The mesylate salt of **1**, marketed as Desferal, is included on the World Health Organization’s list of essential medicines for treating acute iron toxicity and secondary iron overload disease which occurs in blood-transfusion dependent disorders, including β-thalassemia.^5^ Additionally, **1** has a growing presence beyond its primary clinical application with applications as a chelate for radiometal imaging and as a scaffold for siderophore–antibiotic conjugates. Current industrial scale production of **1** is conducted by fermentation, employing *S*. *pilosus*. This results in the coproduction of other siderophores, albeit in far lower concentration, and as such requires rigorous purification. The development of new synthetic approaches toward **1** that might allow diversified production methods would be useful to secure its ongoing use in medicine and the research sector.

**Figure 1:**
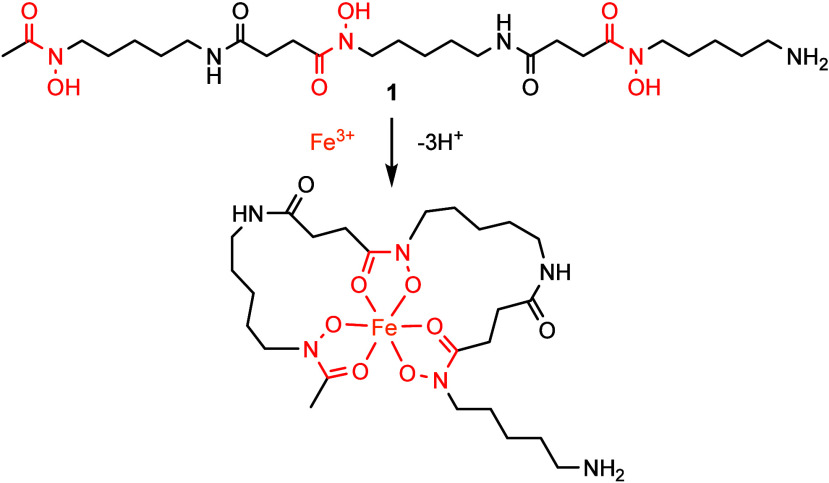
Desferrioxamine B (DFOB) **1** and its iron (Fe^3+^) binding complex.

The first total synthesis of **1** was undertaken in 1962 by Prelog.^8^ This 11-step synthesis generated **1** in 6% overall yield, suffering from low yields in the production of the key starting material, 1-amino-5-nitropentane.^8,9^

Bergeron revisited the total synthesis of **1** in 1988,^10^ which was followed by two further revised pathways, to address some of the limitations of the original attempt, which included production of an unstable cyanobutanal intermediate and inefficient deprotection protocols to liberate the hydroxamic acid and terminal amine groups.^11,12^ More recently, a convergent approach has been used to access **1** as well as other linear and macrocyclic DFO analogues.^13^ Each of these approaches has drawbacks in the use of toxic reagents (e.g., sodium azide), harsh reaction conditions (e.g., Parr hydrogenation), or poor step economy. Described herein is a total synthesis of **1** that overcomes many of these drawbacks by employing a mild installation of the hydroxamic acid moiety and a generalized acylation approach to yield the monomer fragments.

Retrosynthetic analysis (Scheme 1) highlights the initial disconnection of trimeric hydroxamic acid **1** with two amide linkages, readily formed by successive amide coupling reactions.

**Scheme 1:**
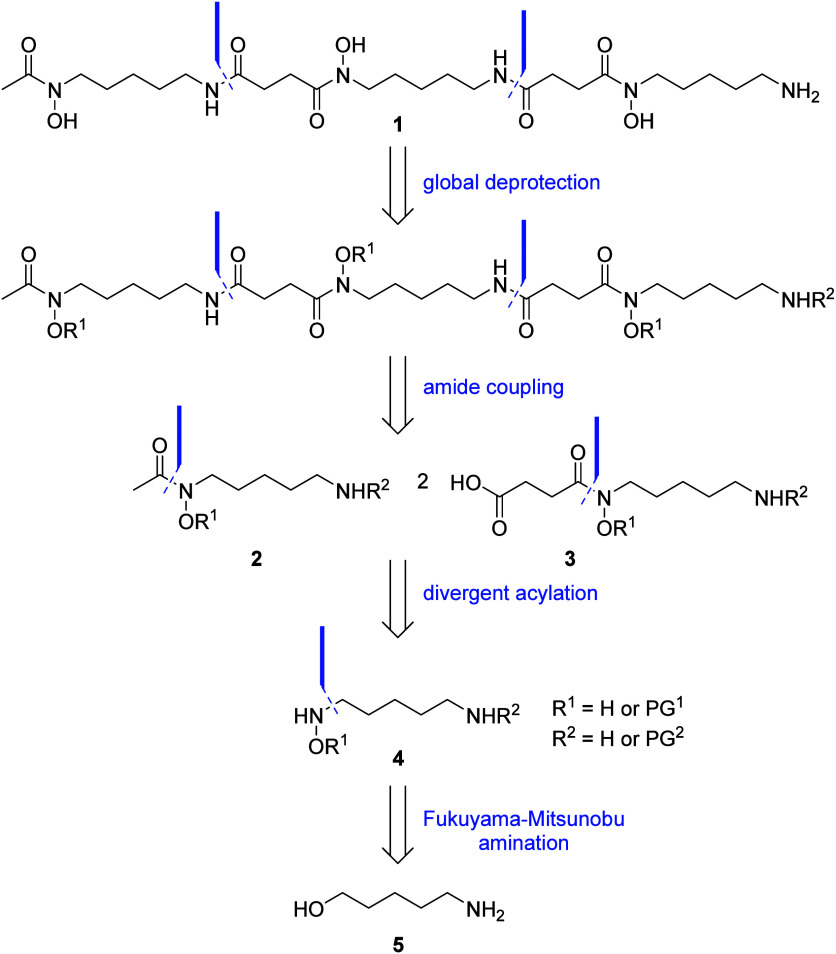
Retrosynthetic Analysis of **1**

This disconnection generates the two natural monomeric species comprising **1**: the blunt-end monomer *N*-hydroxy-*N*-acetylcadaverine (HAC) **2**, and the bifunctional monomer *N*-hydroxy-*N*-succinylcadaverine (HSC) **3**, or protected versions thereof. Both monomers can be generated through a divergent acylation of a common starting material, *N*-hydroxycadaverine **4**. This hydroxamic acid-containing synthon can be generated through a Fukuyama-Mitsunobu amine installation of 5-aminopentan-1-ol **5**, which, in turn, is commercially available at low cost.

This total synthesis of DFOB **1** commenced with the production of two key monomeric fragments from 5-aminopentan-1-ol **5** (Scheme 2). The downstream presence of two nucleophilic amines requires a protecting group strategy to drive regioselective acylation at the hydroxamic acid nitrogen atom. Therefore, the 5-aminopentan-1-ol **5** starting material required protection to mask the reactivity of the primary amine. *N*-Boc-protection of the amine was achieved by treatment of 5-aminopentan-1-ol **5** with di-*tert*-butyl dicarbonate and triethylamine in DCM producing *t*-butyl carbamate **6** in 92% yield. Installation of the key hydroxamic acid has conventionally been achieved by nucleophilic substitution of an alkyl halide precursor with *N*-Boc-protected *O*-benzylhydroxylamine.^12−16^ The major drawbacks of this approach are the use of harsh reaction conditions, namely strong base and high temperature,^11,12,14−16^ and the potential for disubstitution in the case where dihaloalkanes are employed as the starting material.^13^ In addition, where a nitrile substrate is used to generate the protected hydroxamic acid intermediate, harsh reduction conditions are required (Raney Ni, Parr H_2_) to liberate the amine for downstream coupling.^10−12^ A mild alternative to these installations is a Mitsunobu reaction with a mild deprotection of the hydroxamic acid nitrogen.^17−19^ Prior to undertaking the Mitsunobu protocol, *O*-benzylhydroxylamine hydrochloride **7** first required protection to generate a dual protected hydroxylamine reagent **8**.^18−20^ This was achieved by installation of a nosyl unit with 2-nitrobenzylsulfonyl chloride in pyridine, generating **8** in a yield of 70%. The diamine containing substrate **9** was generated by treating the *N*-Boc-protected 5-aminopentan-1-ol **6** with the dual protected hydroxylamine reagent **8** under mild Mitsunobu conditions.^19^ The Mitsunobu product **9** was semipurified by autoflash column chromatography and immediately subjected to nosyl deprotection via the Fukuyama modification. The nosyl group was removed from the diamine by treatment with 2-mercaptoethanol under basic conditions.^19^ This *N*-hydroxycadaverine intermediate **10** was generated in 71% yield over two steps and served as the shared starting material for both monomers comprising **1**.

**Scheme 2:**
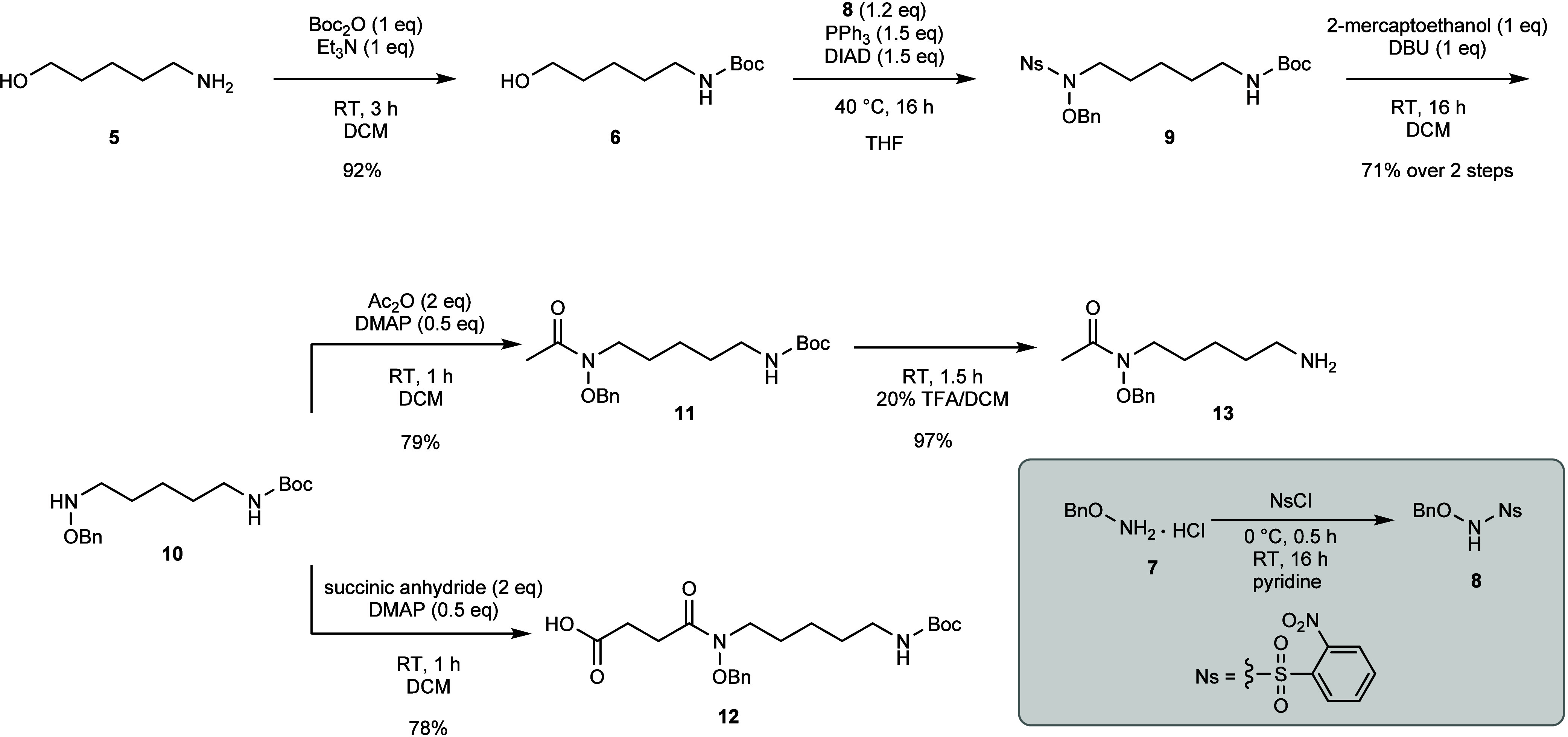
Synthesis of the Two Monomers Comprising DFOB **1**

Previous syntheses of protected versions of the monomeric units, HAC **2** and HSC **3**, have traditionally required long reaction times and in many cases relied upon two distinct protocols for the two substrates rather than a unified approach.^10,12−14,21^ In addition, either one of the monomers has typically been generated through harsher means (i.e., 80–100 °C in DMF or pyridine)^10,13,21^ than the other which often results in laborious workup and purification procedures. In this updated synthesis, a generalized approach was employed which allowed a mild, efficient isolation of both protected HAC **11** and HSC **12** using identical reaction conditions for each of the monomeric units. The protected *N*-hydroxycadaverine **10** was acylated with either acetic anhydride or succinic anhydride, in the presence of substoichiometric amounts of DMAP, to generate protected HAC **11** or HSC **12**, respectively, both in yields of 78–79%. This generalized acylation protocol allowed reactions to occur within 1 h at room temperature, as opposed to overnight at 80 °C, without compromising the yield and purity of either monomer. Subsequent *N*-Boc-deprotection of protected HAC **11** in 20% TFA/DCM liberated the amine **13** in near quantitative amounts, primed for the following amide coupling reaction.

With both monomers in hand, successive amide couplings could be implemented to build the scaffold of **1** (Scheme 3). First, the fully protected HSC monomer **12** was activated with HBTU in DMF in the presence of DIPEA for 30 min before the addition of the amine-deprotected HAC monomer **13**. Following the reaction overnight, the fully protected heterodimer **14** could be readily isolated by preparative HPLC purification in 60% yield. The deprotection-coupling procedure could then be repeated using heterodimer **14**. Specifically, the heterodimer was subjected to the TFA deprotection described earlier to liberate free amine **15** in a nearly quantitative yield of 96%. Subsequent HBTU activation of another equivalent of protected HSC **13** and treatment with the amine-deprotected heterodimer **15** gave fully protected DFOB **16** in 66% yield after purification by preparative HPLC. These amide couplings were also achievable under microwave-assisted conditions giving the coupled products in faster timeframes with a small compromise to yield. The reaction mixtures were irradiated for 10 min at 80 °C giving the protected heterodimer **14** and protected DFOB **16** in moderate yields of 57% and 53% respectively.

**Scheme 3:**
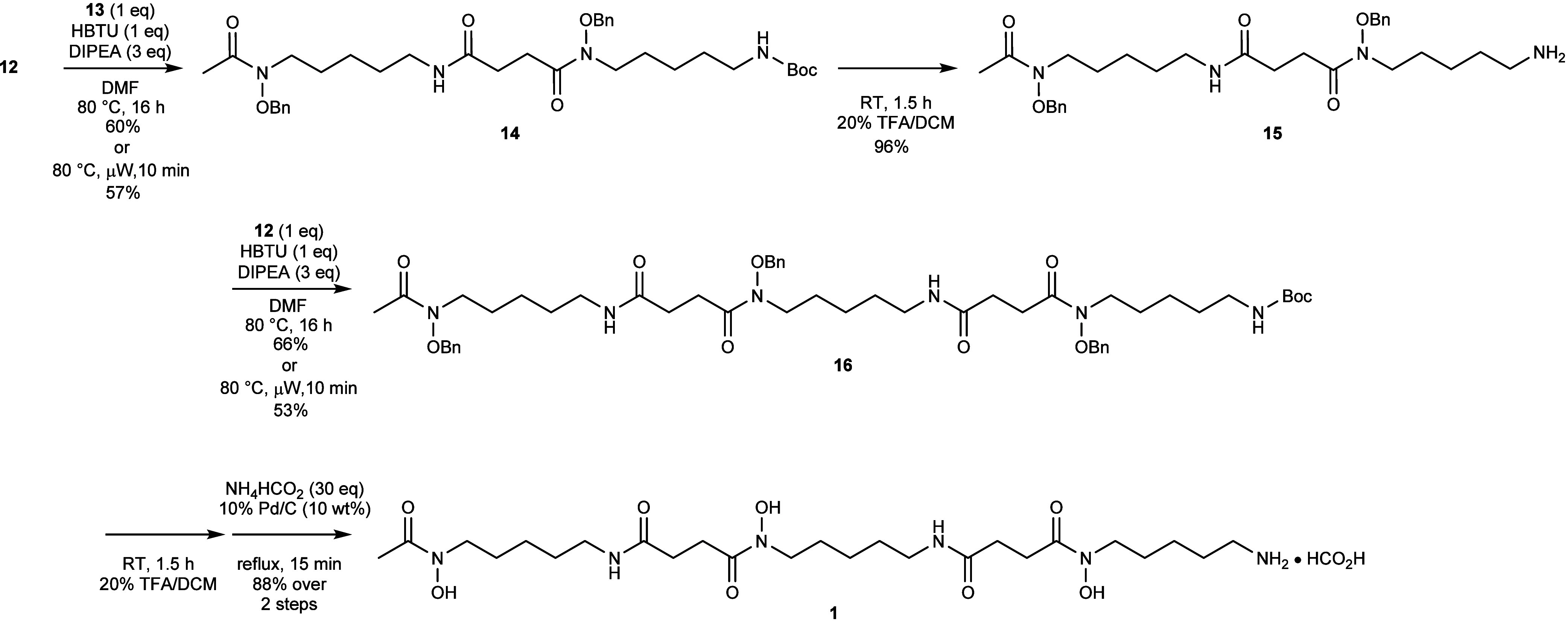
Successive Amide Couplings and Deprotection to Generate **1**

Finally, global deprotection was necessary to liberate the terminal amine group and the hydroxamic acid moieties. The *N*-Boc removal was undertaken using the aforementioned conditions. Subsequent deprotection of the hydroxamic acid moiety has been typically achieved by hydrogenation, with many approaches employing traditional palladium on carbon and hydrogen gas conditions.^8,10−13^ These strategies can lead to decreased yields of the desired hydroxamic acid (≤50%),^13,22^ and/or the isolation of mixtures containing under- and over-reduced byproducts in addition to the desired product.^10,12^

An alternative strategy for debenzylation employs catalytic transfer hydrogenation. These transfer reactions are preferable from a safety standpoint, where instead of hydrogen gas an organic hydrogen donor is employed. Additionally, these transfer strategies provide greater control in terms of selectivity where the reaction parameters can be tailored accordingly. This approach was implemented directly following *N*-Boc-removal to overcome the challenges with traditional hydrogenation strategies. Specifically, the *O*-benzyl protected substrate was treated with excess ammonium formate in a suspension of 10 wt % Pd/C in ethanol at reflux for 15 min. Following removal of Pd/C and lyophilization, **1** was isolated as a formate salt in 88% over the two deprotection steps. The formate salt of DFOB **1** isolated from the total synthesis pathway was found to be in full agreement with the commercially available mesylate of **1** (Figure 2).

**Figure 2:**
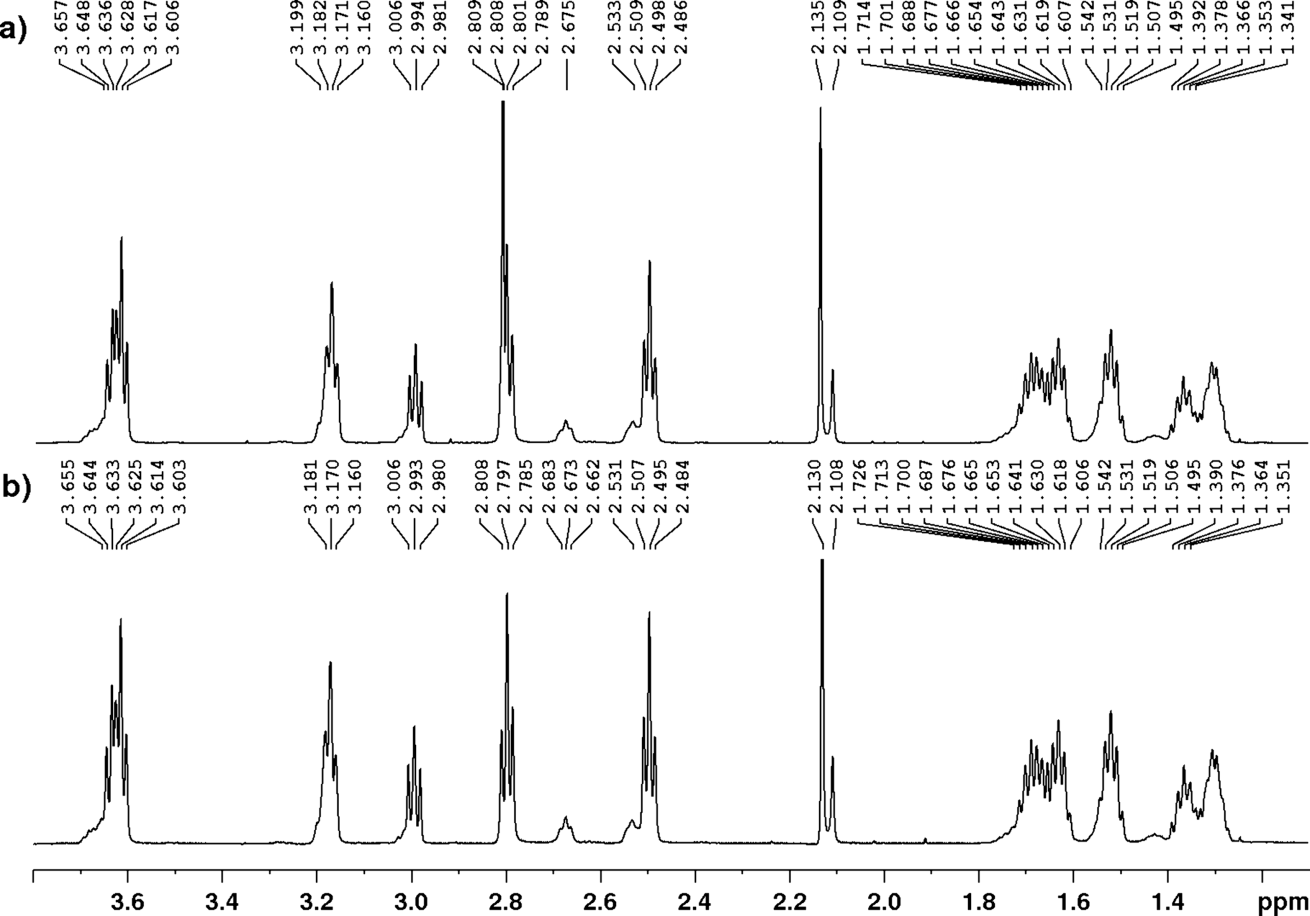
^1^H NMR (600 MHz, D_2_O) spectra of commercially sourced DFOB mesylate **1.Ms** (a) and DFOB formate **1** prepared by total synthesis (b).

Overall, **1** was generated as the formate salt in 17% yield (standard coupling) or 13% yield (microwave-assisted coupling) over a series of ten linear steps. The described total synthesis approach to **1** overcomes many of the drawbacks encountered in previously described approaches. Using 5-aminopentan-1-ol **5** as a starting material with an established amine eliminates the need for selective nitrogen installation of dihaloalkanes,^15,23^ often employing toxic reagents such as sodium azide.^13^ The hydroxamic acid moiety is readily installed by a mild Fukuyama-Mitsunobu amination followed by a divergent acylation to generate both monomeric units **11** and **12** using a standardized, general protocol. Successive HBTU-mediated amide couplings were used to generate the protected DFOB scaffold **16** and could be achieved under standard thermal conditions or microwave-assisted conditions. Subsequent deprotection using standard TFA *N*-Boc removal and a reliable transfer hydrogenation protocol to unmask the hydroxamic acid units, delivered **1** as the formate salt with no observable over-reduction. The efficiency, low-cost starting material, mild conditions, and overall yield of the total synthesis described here could provide an impetus to diversify **1** production methods.

## Experimental Section

### General Methods

Reagents and solvents were purchased from commercial sources and used without further purification, unless otherwise stated. All reactions were heated with an oil bath as the heat source, unless otherwise stated. Thin-layer chromatography was conducted using Chem-Supply silica gel 60 F_254_ TLC plates and visualized under UV light (254 nm) or by ninhydrin stain (1.5% w/v ninhydrin in *n*-butanol with 3% v/v acetic acid). Normal phase autoflash column chromatography was conducted on a Biotage Selekt or Reveleris X2 flash chromatography system using ethyl acetate (EtOAc) and hexane (hex) as eluting solvents with prepacked Biotage Sfar HC or Buchi FlashPure silica cartridges, respectively. Reverse phase autoflash column chromatography was conducted on a Biotage Selekt flash chromatography system using acetonitrile and Milli-Q water as eluting solvents with prepacked Biotage Sfär C18 cartridges. Autoflash column chromatography solvent programs are expressed in column volumes (CV) and are stated below for each compound.

Preparative HPLC purification was undertaken on a Shimadzu LC-20 series high performance liquid chromatography system with two LC-20AP preparative pumps, a SIL-10AP autosampler, and a SPD-20A photodiode array detector. A Shim-pack GIS-C18 column (150 mm × 20 ID, 5 μm) was used at 20 mL/min. Mobile phase A was 0.1% formic acid in Milli-Q water, and mobile phase B was 0.1% formic acid in acetonitrile with solvent programs expressed in time as stated below for individual compounds.

^1^H and ^13^C NMR spectra were acquired on a 600 MHz Bruker Avance III NMR spectrometer equipped with a TCI cryoprobe at 600 and 150 MHz, respectively. Spectra were recorded at 298 K using CDCl_3_, DMSO-*d*_6_, or D_2_O as the solvent and internal lock. All spectra were referenced to the residual solvent signal (CDCl_3_: ^1^H 7.26 ppm, ^13^C 77.0 ppm; DMSO-*d*_6_: ^1^H 2.50 ppm, ^13^C 39.52 ppm, D_2_O: ^1^H: 4.79 ppm) and are reported as follows: (1) chemical shift (ppm), (2) integration, (3), multiplicity (s, singlet; brs, broad singlet; d, doublet; dd, doublet of doublets; t, triplet; m, multiplet); and (4) coupling constant (Hz).

HRMS analysis was conducted on a Thermofisher Vanquish Horizon UHPLC instrument coupled to a Thermofisher Q-Exactive HFX Hybrid Quadrupole-Orbitrap mass spectrometer. Solvent A was 0.1% formic acid in water, and solvent B was 0.1% formic acid in acetonitrile. An Agilent Zorbax Eclipse XDB-C18 column (150 mm × 2.1 mm I.D., 3.5 μm) maintained at 30 °C with a constant flow rate of 0.2 mL/min was employed. A sample injection volume of 5 μL was used, and the solvent program was as follows: a linear gradient of 5–95% solvent B over 25 min followed by 95% solvent B held for 5 min. Solvent B was then held at 5% for an additional 5 min to re-equilibrate for the following injection. The mass spectrometer was operated in positive ion-mode using electrospray ionization (ESI) with a mass range of 100–1500 *m*/*z*. A spray voltage of 3.5 kV was used, and the capillary temperature was maintained at 300 °C.

Microwave reactions were conducted in a CEM Discover-SP microwave reactor using 10 or 35 mL sealed reaction vessels filled. The reactor was operated in dynamic mode with a power limit of 150 W and high levels of stirring. Reactions were set to a required temperature and, following completion, were cooled with gas jet cooling.

### Synthetic Protocols

#### *tert*-Butyl (5-hydroxypentyl)carbamate **6**(19)

A solution of di-*tert*-butyl dicarbonate (6.52 g, 29.9 mmol) in dichloromethane (25 mL) was slowly added to a solution of 5-aminopentan-1-ol 5 (3.05 g, 29.6 mmol) in dichloromethane (25 mL). Triethylamine (4.20 mL, 30.1 mmol) was added and the resulting solution was left to stir at room temperature for 3 h. The solvent was removed *in vacuo* and the residue purified by autoflash column chromatography (Biotage; 1 CV: 5% EtOAc in hex., 10 CV: 5–40% EtOAc in hex, 9 CV: 40% EtOAc in hex) to give *N*-Boc-5-aminopentan-1-ol **6** (5.54 g, 92%) as a colorless oil.

^1^H NMR (600 MHz, CDCl_3_) δ 4.57 (1H, brs), 3.63 (2H, dd, *J* = 6.4, 11.9 Hz), 3.12 (2H, dd, *J* = 5.9, 12.2 Hz), 1.58 (2H, m), 1.50 (2H, m), 1.43 (9H, brs), 1.37 (2H, m). ^13^C{^1^H} (150 MHz, CDCl_3_) δ: 156.0, 79.1, 62.6, 40.4, 32.2, 29.8, 28.4, 22.9. HRMS (ESI) *m*/*z*: [M + H]^+^ calcd for C_10_H_22_NO_3_ 204.1594; found 204.1595.

#### *N*-(Benzyloxy)-2-nitrobenzenesulfonamide **8**(19)

A solution of 2-nitrobenzylsulfonyl chloride (10.7 g, 48.3 mmol) in pyridine (50 mL) was added dropwise to a stirring solution of *O*-benzylhydroxylamine hydrochloride **7** (7.5 g, 47.0 mmol) in pyridine (90 mL) at 0 °C. Following complete addition, the reaction mixture was stirred for an additional 30 min at 0 °C before being warmed to room temperature. The mixture was left to stir at room temperature overnight. The reaction was quenched with water (30 mL) and the mixture was concentrated *in vacuo*. The resulting aqueous mixture was further diluted with water (100 mL) and extracted with ethyl acetate (3 × 50 mL). The ethyl acetate fraction was washed sequentially with hydrochloric acid (1 M, 3 × 20 mL), water (3 × 20 mL), and saturated sodium bicarbonate solution (3 × 20 mL). The ethyl acetate fraction was dried over sodium sulfate, and the solvent was removed *in vacuo* to give *N*-(benzyloxy)-2-nitrobenzenesulfonamide **8** (10.1 g, 70%) as a brown solid that was used without further purification.

^1^H NMR (600 MHz, CDCl_3_) δ: 8.25 (1H, m), 8.12 (1H, brs), 7.87 (1H, m), 7.78 (2H, m), 7.98–7.34 (5H, m), 5.07 (2H, s). ^13^C{^1^H} (150 MHz, CDCl_3_) δ: 148.4, 134.8, 134.7, 133.7, 132.8, 130.3, 129.4, 128.9, 128.6, 125.5, 79.8.

#### *tert*-Butyl (5-((benzyloxy)amino)pentyl)carbamate **10**(19)

Triphenylphosphine (9.75 g, 37.2 mmol) and *N*-(benzyloxy)-2-nitrobenzenesulfonamide **8** (9.16 g, 29.7 mmol) were added to a solution of *N*-Boc-5-aminopentan-1-ol **6** (5.00 g, 24.6 mmol) in THF (60 mL). The mixture was stirred at room temperature for 15 min to ensure complete dissolution. The resulting solution was cooled to 0 °C and DIAD (7.50 mL, 38.1 mmol) was added dropwise. Following complete addition, the resulting mixture was stirred at 0 °C for 30 min. The reaction mixture was then warmed to 40 °C and left to stir overnight. The resulting solution was concentrated *in vacuo* and the crude material reconstituted with diethyl ether (50 mL). The resulting mixture was stirred at 0 °C over an ice bath, and the resulting precipitate was removed by filtration. The solvent was removed *in vacuo* and the crude material as **9** was semipurified by autoflash column chromatography (Reveleris; 1.5 CV: 9% EtOAc in hex, 16 CV: 9–28% EtOAc in hex, 3 CV: 28% EtOAc in hex).

The semipurified material was reconstituted in DMF (25 mL) and 2-mercaptoethanol (1.75 mL, 24.9 mmol) and DBU (3.70 mL, 24.7 mmol) were added. The resulting mixture was stirred at room temperature overnight. The solvent was then removed *in vacuo* and the crude material reconstituted in dichloromethane. The dichloromethane solution was thoroughly washed with water and dried with sodium sulfate. The solvent was removed *in vacuo* and the crude material purified by autoflash column chromatography (Biotage; 1 CV: 15% EtOAc in hex, 25 CV: 15–40% EtOAc in hex, 10 CV: 40% EtOAc in hex) giving *tert*-butyl (5-((benzyloxy)amino)pentyl)carbamate **10** (5.48 g, 71%) as a yellow oil.

^1^H NMR (600 MHz, CDCl_3_) δ 7.35–7.28 (5H, m), 5.52 (1H, brs), 4.69 (2H, s), 3.10 (2H, m), 2.92 (2H, t, *J* = 7.1 Hz), 1.53 (2H, m), 1.46 (2H, m), 1.43 (9H, s), 1.33 (2H, m). ^13^C{^1^H} (150 MHz, CDCl_3_) δ: 156.0, 138.0, 128.4, 127.8, 76.2, 52.0, 40.5, 30.0, 28.4, 27.1, 24.4, 22.0. HRMS (ESI) *m*/*z*: [M + H]^+^ calcd for C_17_H_29_N_2_O_3_ 309.2173; found 309.2170.

### General Acylation Procedure

Adapted from Chiu,^13^ DMAP (0.5 equiv) and an appropriate anhydride (2 equiv) were added to a solution of *tert*-butyl (5-((benzyloxy)amino)pentyl)carbamate **10** (1 equiv) in dichloromethane (0.3 M). The resulting mixture was allowed to stir at room temperature for 1 h before being diluted with dichloromethane (0.1 M). The solution was washed with hydrochloric acid (1 M) and the DCM fraction was dried over sodium sulfate. The solvent was removed *in vacuo* and the crude material purified by autoflash column chromatography (Biotage; 2 CV: 35% EtOAc in hex, 15 CV: 35–65% EtOAc in hex, 6 CV: 65% EtOAc in hex) to yield the desired protected hydroxamic acid.

#### *tert*-Butyl (5-(*N*-(benzyloxy)acetamido)pentyl)carbamate **11**

*tert*-Butyl (5-(*N*-(benzyloxy)acetamido)pentyl)carbamate **11** was prepared using the general acylation procedure using DMAP (308 mg, 2.52 mmol), acetic anhydride (1 mL, 10.6 mmol), and *tert*-butyl (5-((benzyloxy)amino)pentyl)carbamate **10** (1.62 g, 5.25 mmol). *tert*-Butyl (5-(*N*-(benzyloxy)acetamido)pentyl)carbamate **11** (1.46 g, 79%) was isolated as a colorless oil.

^1^H NMR (600 MHz, DMSO-*d*_6_) δ 7.41–7.36 (5H, m), 6.75 (1H, t, *J* = 5.3 Hz), 4.86 (2H, s), 3.56 (2H, t, *J* = 6.9 Hz), 2.87 (2H, dd, *J* = 6.7, 12.9 Hz), 1.99 (3H, s), 1.51 (2H, m), 1.36 (9H, s), 1.34 (2H, m), 1.19 (2H, m). ^13^C{^1^H} (150 MHz, DMSO-*d*_6_) δ: 171.0, 155.6, 134.9, 129.4, 128.7, 128.5, 77.3, 75.3, 29.1, 28.3, 26.2, 23.4, 20.3. HRMS (ESI) *m*/*z*: [M + H]^+^ calcd for C_19_H_31_N_2_O_4_ 351.2278; found 351.2278.

#### 4-((Benzyloxy)(5-((*tert*-butoxycarbonyl)amino)pentyl)amino)-4-oxobutanoic acid **12**(11)

4-((Benzyloxy)(5-((*tert*-butoxycarbonyl)amino)pentyl)amino)-4-oxobutanoic acid **12** was prepared using the general acylation procedure using DMAP (405 mg, 3.32 mmol), succinic anhydride (1.33 g, 13.3 mmol), and *tert*-butyl (5-((benzyloxy)amino)pentyl)carbamate **10** (2.04 g, 6.61 mmol). 4-((Benzyloxy)(5-((*tert*-butoxycarbonyl)amino)pentyl)amino)-4-oxobutanoic acid **12** (2.1 g, 78%) was isolated as a white solid.

^1^H NMR (600 MHz, DMSO-*d*_6_) δ 7.44–7.37 (5H, m) 6.74 (1H, t, *J* = 5.4 Hz), 4.88 (2H, s), 3.56 (2H, t, *J* = 6.9 Hz), 2.87 (2H, dd, *J* = 6.7, 12.9 Hz), 2.62 (2H, m), 2.42 (2H, t, *J* = 6.9 Hz), 1.51 (2H, m), 1.36 (9H, s), 1.33 (2H, m), 1.18 (2H, m). ^13^C{^1^H} (150 MHz, DMSO-*d*_6_) δ: 173.8, 155.6, 134.9, 129.3, 128.7, 128.5, 77.3, 75.4, 29.1, 28.3, 28.3, 26.8, 26.1, 23.4. HRMS (ESI) *m*/*z*: [M + H]^+^ calcd for C_21_H_33_N_2_O_6_ 409.2333; found 409.2330.

#### *N*-(5-Aminopentyl)-*N*-(benzyloxy)acetamide **13**

*tert*-Butyl (5-(*N*-(benzyloxy)acetamido)pentyl)carbamate **11** (1.49 g, 4.25 mmol) was stirred in TFA/DCM (20% v/v, 10 mL) for 1.5 h. The residue was reconstituted, and the solvent removed *in vacuo* in ethyl acetate (3 × 10 mL) followed by toluene (3 × 10 mL). The resulting material was purified by reverse-phase autoflash column chromatography (Biotage; 6 CV: Milli-Q H_2_O, 6 CV: 80% MeCN in Milli-Q H_2_O) and lyophilized to give the deprotected amine **13** (1.03 g, 97%) as a colorless oil.

^1^H NMR (600 MHz, DMSO-*d*_6_) δ 7.42–7.39 (5H, m), 4.87 (2H, s), 3.59 (2H, t, *J* = 6.9 Hz), 2.75 (2H, m), 2.01 (3H, s), 1.53 (4H, m), 1.26 (2H, m). ^13^C{^1^H} (150 MHz, DMSO-*d*_6_) δ: 171.0, 134.9, 129.4, 128.7, 128.5, 75.3, 44.2, 41.4, 32.8, 28.9, 26.4, 23.6, 20.3. HRMS (ESI) *m*/*z*: [M + H]^+^ calcd for C_14_H_23_N_2_O_2_ 251.1754; found 251.1751.

#### *tert*-Butyl (3-acetyl-14-(benzyloxy)-10,13-dioxo-1-phenyl-2-oxa-3,9,14-triazanonadecan-19-yl)carbamate **14**

HBTU (1.29 g, 3.40 mmol) and DIPEA (1.75 mL, 10.0 mmol) were added to a solution of 4-((benzyloxy)(5-((*tert*-butoxycarbonyl)amino)pentyl)amino)-4-oxobutanoic acid **12** (1.34 g, 3.28 mmol) in anhydrous DMF (10 mL) under an atmosphere of nitrogen. The resulting mixture was left to stir at room temperature for 0.5 h. *N*-(5-Aminopentyl)-*N*-(benzyloxy)acetamide **13** (823 mg, 3.29 mmol) in anhydrous DMF (10 mL) was added to the mixture and heated to 80 °C. Following continued heating overnight, the solvent was removed *in vacuo* and the crude material reconstituted in dichloromethane (100 mL). The dichloromethane solution was sequentially washed with a saturated sodium bicarbonate solution (2 × 50 mL) and water (2 × 50 mL). The dichloromethane fraction was dried over sodium sulfate and the solvent removed *in vacuo*. The crude material was purified by preparative HPLC (60–66% solvent B over 10 min) and lyophilized to give the heterodimer **14** (1.26 g, 60%) as a pale, yellow oil.

HBTU (843 mg, 2.22 mmol) and DIPEA (1.2 mL, 6.89 mmol) were added to a solution of 4-((benzyloxy)(5-((*tert*-butoxycarbonyl)amino)pentyl)amino)-4-oxobutanoic acid **12** (904 mg, 2.21 mmol) in anhydrous DMF (4 mL). *N*-(5-Aminopentyl)-*N*-(benzyloxy)acetamide **13** (554 mg, 2.21 mmol) in anhydrous DMF (4 mL) was added to the mixture, and the reaction vessel was sealed. The reaction mixture was subjected to microwave irradiation at 80 °C and power limit of 150 W for 10 min. The reaction was cooled to 50 °C by gas jet cooling. The solvent was removed *in vacuo*, and the crude material was reconstituted in dichloromethane (15 mL). The dichloromethane solution was sequentially washed with saturated sodium bicarbonate solution (2 × 10 mL) and water (2 × 10 mL). The dichloromethane fraction was dried over sodium sulfate and the solvent removed *in vacuo*. The crude material was purified by preparative HPLC (60–66% solvent B over 10 min) and lyophilized to give the heterodimer **14** (808 mg, 57%) as a pale, yellow oil.

^1^H NMR (600 MHz, DMSO-*d*_6_) δ 7.77 (1H, t, *J* = 5.4 Hz), 7.44–7.36 (10H, m), 6.75 (1H, t, *J* = 5.7 Hz), 4.87 (2H, s), 4.86 (2H, s), 3.56 (4H, dd, *J* = 7.2, 14.5 Hz), 2.99 (2H, dd, *J* = 6.8, 12.8 Hz), 2.87 (2H, dd, *J* = 6.7, 13.1 Hz), 2.61 (2H, m), 2.29 (2H, t, *J* = 7.2 Hz), 1.99 (3H, s), 1.51 (4H, m), 1.36 (13H, m), 1.20 (4H, m). ^13^C{^1^H} (150 MHz, DMSO-*d*_6_) δ: 172.8, 170.9, 163.0, 155.5, 134.9, 129.4, 129.2, 128.6, 128.5, 128.4, 77.3, 75.4, 75.3, 44.44, 44.1, 38.7, 29.1, 28.7, 28.2, 27.2, 26.1, 23.5, 23.4, 20.3. HRMS (ESI) *m*/*z*: [M + H]^+^ calcd for C_35_H_53_N_4_O_7_ 641.3909; found 641.3905.

#### *N*^1^-(5-Aminopentyl)-*N*^1^-(benzyloxy)-*N*^4^-(5-(*N*-(benzyloxy)acetamido)pentyl)succinimide **15**

*tert*-Butyl (3-acetyl-14-(benzyloxy)-10,13-dioxo-1-phenyl-2-oxa-3,9,14-triazanonadecan-19-yl)carbamate **14** (507 mg, 0.791 mmol) was stirred in TFA/DCM (20% v/v, mL) for 1.5 h. The residue was reconstituted, and the solvent removed *in vacuo* in ethyl acetate (3 × 10 mL) followed by toluene (3 × 10 mL). The resulting material was purified by reverse-phase autoflash column chromatography (Biotage; 6 CV: Milli-Q H_2_O, 6 CV: 80% MeCN in Milli-Q H_2_O) and lyophilized to give the deprotected amine **15** (410 mg, 96%) as a colorless oil.

^1^H NMR (600 MHz, DMSO-*d*_6_) δ 7.79 (1H, t, *J* = 5.5 Hz), 7.44–7.37 (10H, m), 4.88 (2H, s), 4.86 (2H, m), 3.57 (4H, m), 2.98 (2H, dd, *J* = 6.8, 12.7 Hz), 2.73 (2H,), 2.62 (2H, m), 2.31 (2H, t, *J* = 7.0 Hz), 1.99 (3H, s), 1.52 (6H, m), 1.37 (2H, m), 1.21 (4H, m). ^13^C{^1^H} (150 MHz, DMSO-*d*_6_) δ: 172.9, 171.0, 134.9, 129.4, 129.3, 128.6, 128.5, 75.4, 75.3, 44.3, 44.1, 40.2, 38.4, 29.7, 28.8, 28.0, 27.2, 26.2, 23.5, 23.3, 20.3. HRMS (ESI) *m*/*z*: [M + H]^+^ calcd for C_30_H_45_N_4_O_5_ 541.3384; found 541.3386.

#### *tert*-Butyl (3-acetyl-14,25-bis(benzyloxy)-10,13,21,24-tetraoxo-1-phenyl-2-oxa-3,9,14,20,25-pentaazatriacontan-30-yl)carbamate **16**

HBTU (110 mg, 0.29 mmol) and DIPEA (140 μL, 0.80 mmol) were added to a solution of 4-((benzyloxy)(5-((*tert*-butoxycarbonyl)amino)pentyl)amino)-4-oxobutanoic acid **12** (112 mg, 0.27 mmol) in anhydrous DMF (2 mL) under an atmosphere of nitrogen. The resulting mixture was left to stir at room temperature for 0.5 h. *N*^1^-(5-Aminopentyl)-*N*^1^-(benzyloxy)-*N*^4^-(5-(*N*-(benzyloxy)acetamido)pentyl)succinimide **15** (142 mg, 0.26 mmol) in anhydrous DMF (2 mL) was added to the mixture and heated to 80 °C. Following continued heating overnight, the solvent was removed *in vacuo* and the crude material reconstituted in dichloromethane (20 mL). The dichloromethane solution was sequentially washed with saturated sodium bicarbonate solution (2 × 10 mL) and water (2 × 10 mL). The dichloromethane fraction was dried over sodium sulfate and the solvent removed *in vacuo*. The crude material was purified by preparative HPLC (65% solvent B held for 2 min, 65–80% solvent B over 10 min) and lyophilized to give protected DFOB **16** (169 mg, 66%) as a pale, yellow oil.

HBTU (198 mg, 0.52 mmol) and DIPEA (260 μL, 1.49 mmol) were added to a solution of 4-((benzyloxy)(5-((*tert*-butoxycarbonyl)amino)pentyl)amino)-4-oxobutanoic acid **12** (200 mg, 0.49 mmol) in anhydrous DMF (1.5 mL). *N*^1^-(5-Aminopentyl)-*N*^1^-(benzyloxy)-*N*^4^-(5-(N-(benzyloxy)acetamido)pentyl)succinimide **15** (250 mg, 0.46 mmol) in anhydrous DMF (1.5 mL) was added to the mixture, and the reaction vessel was sealed. The reaction mixture was subjected to microwave irradiation at 80 °C and power limit of 150 W for 10 min. The reaction was cooled to 50 °C by gas jet cooling. The solvent was removed *in vacuo*, and the crude material was reconstituted in dichloromethane (10 mL). The dichloromethane solution was sequentially washed with saturated sodium bicarbonate solution (2 × 5 mL) and water (2 × 5 mL). The dichloromethane fraction was dried over sodium sulfate and the solvent removed *in vacuo*. The crude material was purified by preparative HPLC (60–66% solvent B over 10 min) and lyophilized to give protected DFOB **16** (228 mg, 53%) as a pale, yellow oil.

^1^H NMR (600 MHz, DMSO-*d*_6_) δ 7.76 (2H, m), 7.43–7.35 (15H, m), 6.74 (1H, t, *J* = 5.4 Hz), 4.87 (4H, brs), 4.85 (2H, s), 3.55 (6H, m), 2.98 (4H, m), 2.87 (2H, dd, *J* = 6.7, 13.0 Hz), 2.62 (4H, m), 2.29 (4H, t, *J* = 7.0 Hz), 1.99 (3H, s), 1.50 (6H, m), 1.35 (15H, m), 1.20 (6H, m). ^13^C{^1^H} (150 MHz, DMSO-*d*_6_) δ: 172.9, 171.0, 170.9, 155.6, 134.9, 129.4, 129.3, 128.6, 128.5, 77.3, 75.4, 75.3, 44.4, 44.1, 40.0, 38.4, 29.7, 29.1, 28.7, 28.3, 27.2, 26.1, 23.5, 23.4. HRMS (ESI) *m*/*z*: [M + H]^+^ calcd for C_51_H_75_N_6_O_10_ 931.5539; found 931.5522.

#### *N*^1^-(5-Aminopentyl)-*N*^1^-hydroxy-*N*^4^-(5-(*N*-hydroxy-4-((5-(*N*-hydroxyacetamido)pentyl)amino)-4-oxobutanamido)pentyl)succinimide, DFOB **1**

*tert*-Butyl (3-acetyl-14,25-bis(benzyloxy)-10,13,21,24-tetraoxo-1-phenyl-2-oxa-3,9,14,20,25-pentaazatriacontan-30-yl)carbamate **16** (140 mg, 0.15 mmol) was stirred in TFA/DCM (20% v/v, mL) for 1.5 h. The residue was reconstituted, and the solvent removed *in vacuo* in ethyl acetate (3 × 10 mL) followed by toluene (3 × 10 mL). The resulting material was purified by reverse-phase autoflash column chromatography (Biotage; 3 CV: Milli-Q H_2_O, 6 CV: 80% MeCN in Milli-Q H_2_O) to give the deprotected amine. The resulting free amine in ethanol (2 mL) was added to a suspension of Pd/C (12 mg, 10 wt %) in ethanol (3 mL). Ammonium formate (249 mg, 3.94 mmol) was added to the mixture and was heated at reflux under nitrogen for 15 min. The reaction mixture was cooled and filtered over Celite and the filtrate concentrated *in vacuo*. The material was reconstituted in water and lyophilized to give DFOB **1** (80 mg, 88%) as a formate salt. NOTE: DFOB **1** exists as a mixture of *E/Z* conformations about the terminal acetyl hydroxamate in D_2_O.^11^

^1^H NMR (600 MHz, D_2_O) δ 3.64 (6H, m), 3.17 (4H, m), 2.99 (2H,), 2.79 (3.2H, t, *J* = Hz), 2.67(0.8H, m), 2.53 (0.8H, m), 2.49 (3.2H, t, *J* = Hz), 2.13 (2H, s), 2.11 (1H, s), 1.69 (4H, m), 1.63 (4H, m), 1.52 (4H, m), 1.36 (2H, m), 1.30 (4H, m). ^13^C{^1^H} (150 MHz, D_2_O) δ: 174.4, 173.2, 173.1, 172.8, 170.5, 47.4, 47.3, 47.2, 38.9, 38.7, 30.0, 29.9, 27.4, 27.3, 27.1, 27.0, 25.8, 25.6, 25.0, 24.8, 22.6, 22.2, 19.1, 18.7. HRMS (ESI) *m*/*z*: [M + H]^+^ calcd for C_25_H_49_N_6_O_8_ 561.3606; found 561.3620.

## Data Availability

The data underlying this study are available in the published article and its Supporting Information.

## Supporting Information Available

^1^H and ^13^C NMR spectra (PDF)
