# Supplementary material for: A Mild and Modular Approach to the Total Synthesis of Desferrioxamine B
Source: J Org Chem. 2024 Mar 12;89(7):5118–25. doi: 10.1021/acs.joc.3c02739 (PMC11003418; doi:10.1021/acs.joc.3c02739)
Supplement: Supplementary file 1 — jo3c02739_si_001.pdf [file jo3c02739_si_001.pdf]

Supporting Information

A Mild and Modular Approach to the Total Synthesis of Desferrioxamine B

Todd E. Markham and Rachel Codd*

School of Medical Sciences, The University of Sydney, Sydney, New South Wales 2006, Australia.

e-mail: rachel.codd@sydney.edu.au

Table of Contents

Item	Pg
^{1}H and ^{13}C NMR spectra of **6**	S2
^{1}H and ^{13}C NMR spectra of **8**	S3
^{1}H and ^{13}C NMR spectra of **10**	S4
^{1}H and ^{13}C NMR spectra of **11**	S5
^{1}H and ^{13}C NMR spectra of **12**	S6
^{1}H and ^{13}C NMR spectra of **13**	S7
^{1}H and ^{13}C NMR spectra of **14**	S8
^{1}H and ^{13}C NMR spectra of **15**	S9
^{1}H and ^{13}C NMR spectra of **16**	S10
^{1}H and ^{13}C NMR spectra of **1**	S11

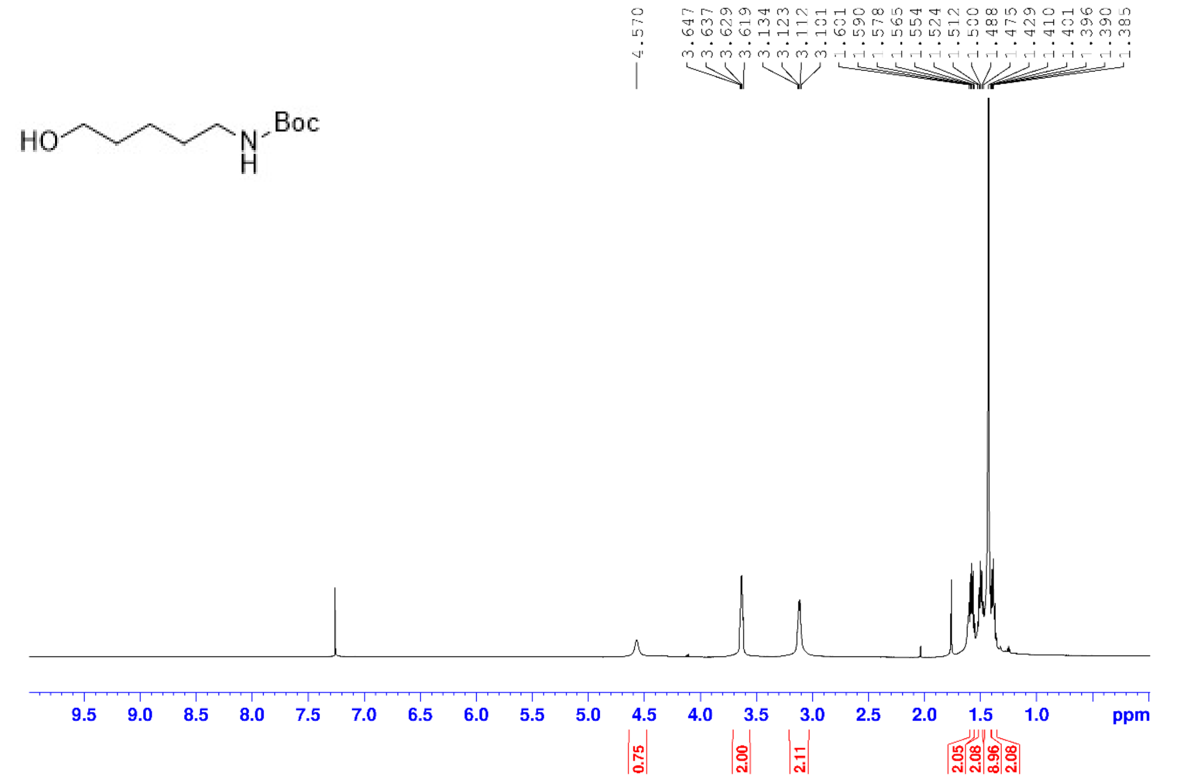

Figure S1. ^{1}H NMR (600 MHz, $CDCl_3$) spectrum of **6**

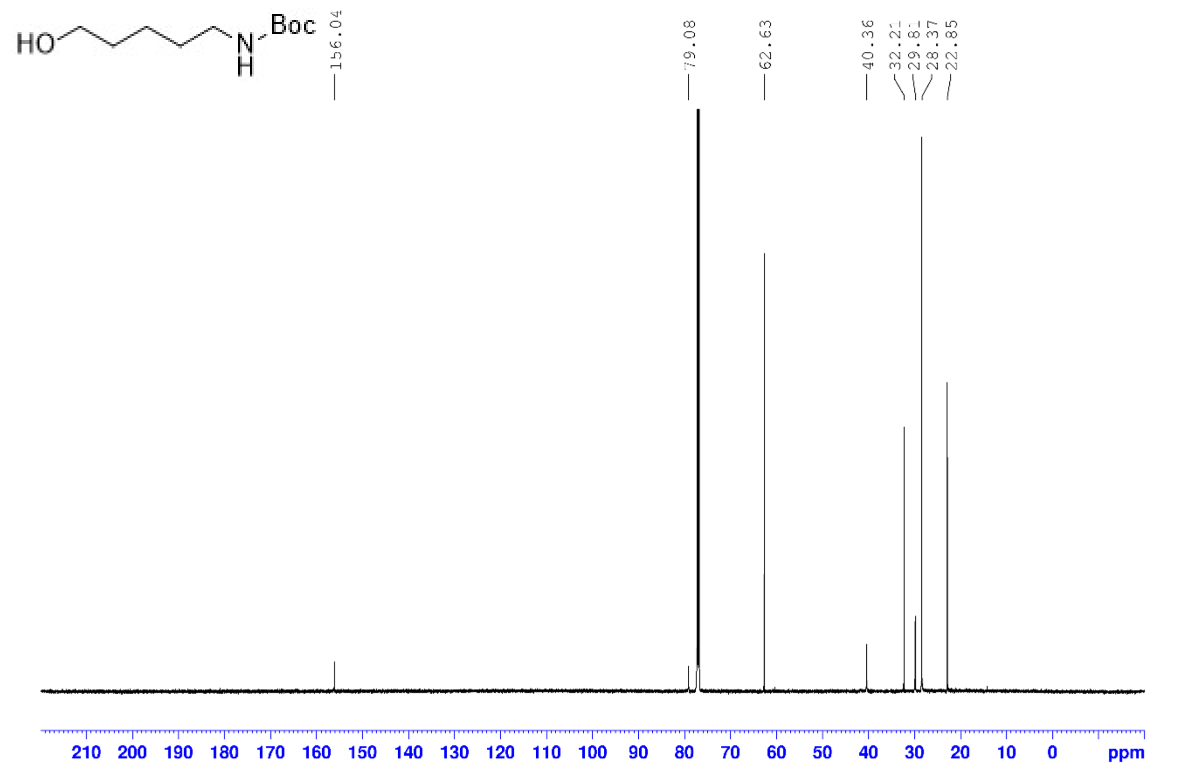

Figure S2. $^{13}C\{^{1}H\}$ NMR (150 MHz, $CDCl_3$) spectrum of **6**

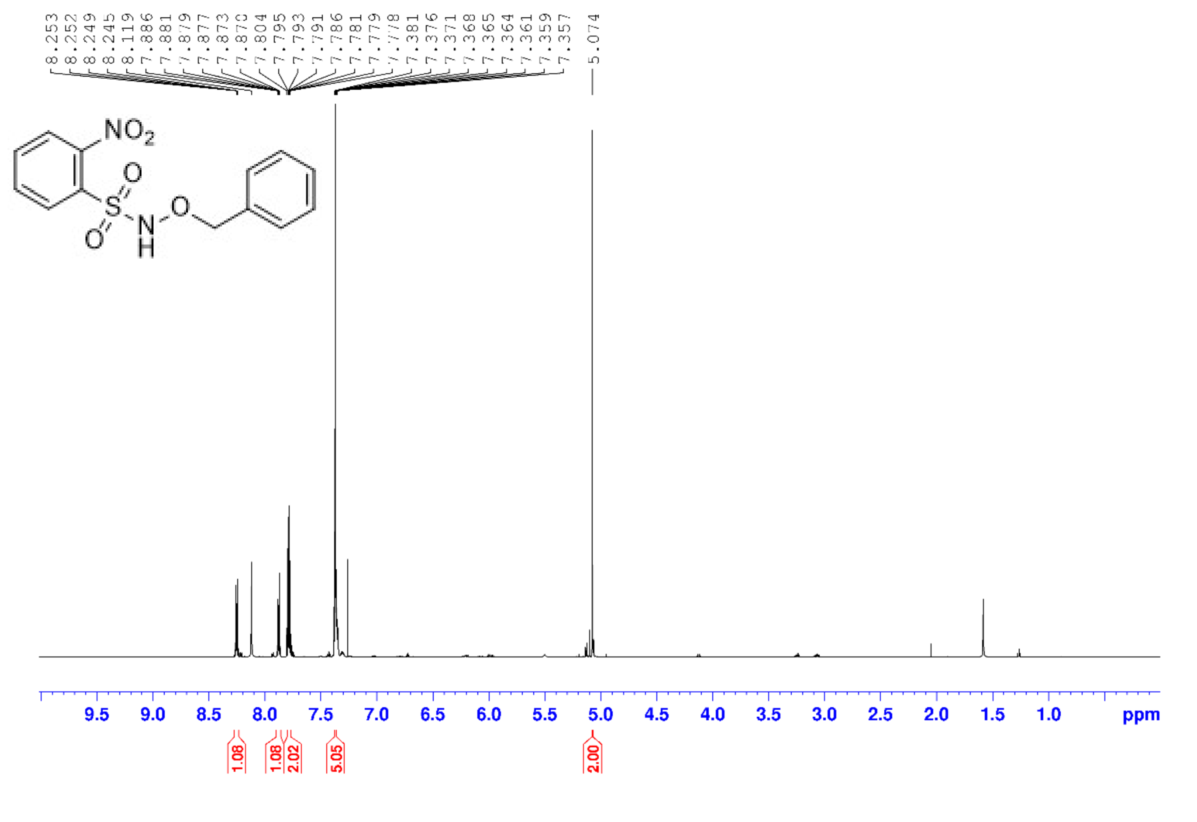

Figure S3. ^{1}H NMR (600 MHz, $CDCl_3$) spectrum of **8**

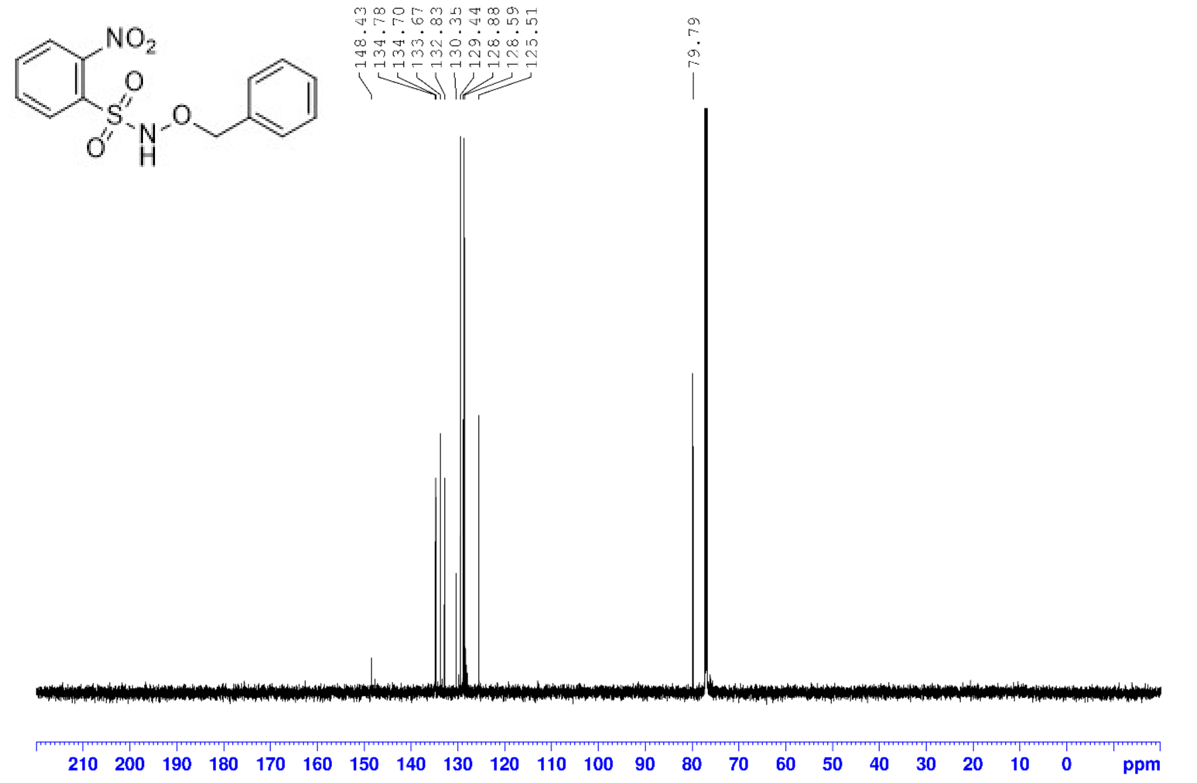

Figure S4. $^{13}C\{^{1}H\}$ NMR (150 MHz, $CDCl_3$) spectrum of **8**

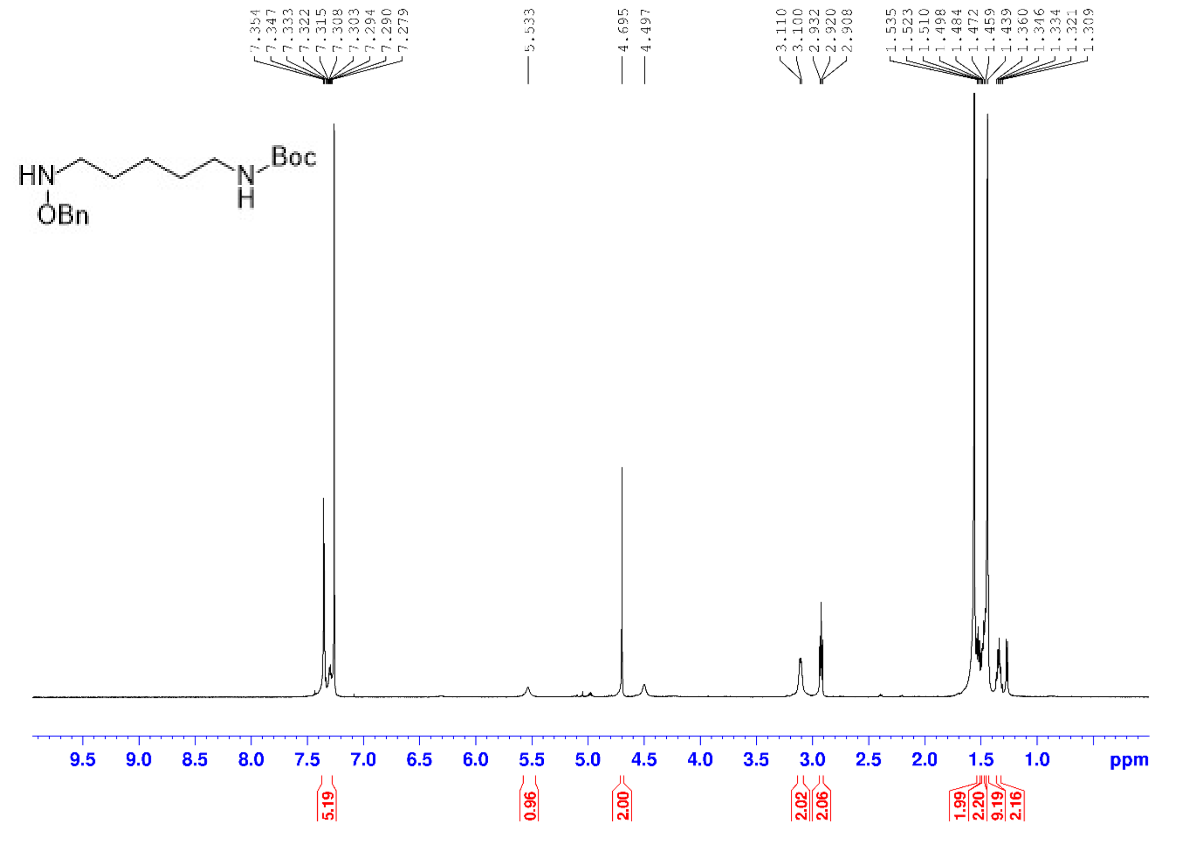

Figure S5. ^{1}H NMR (600 MHz, $CDCl_3$) spectrum of **10**

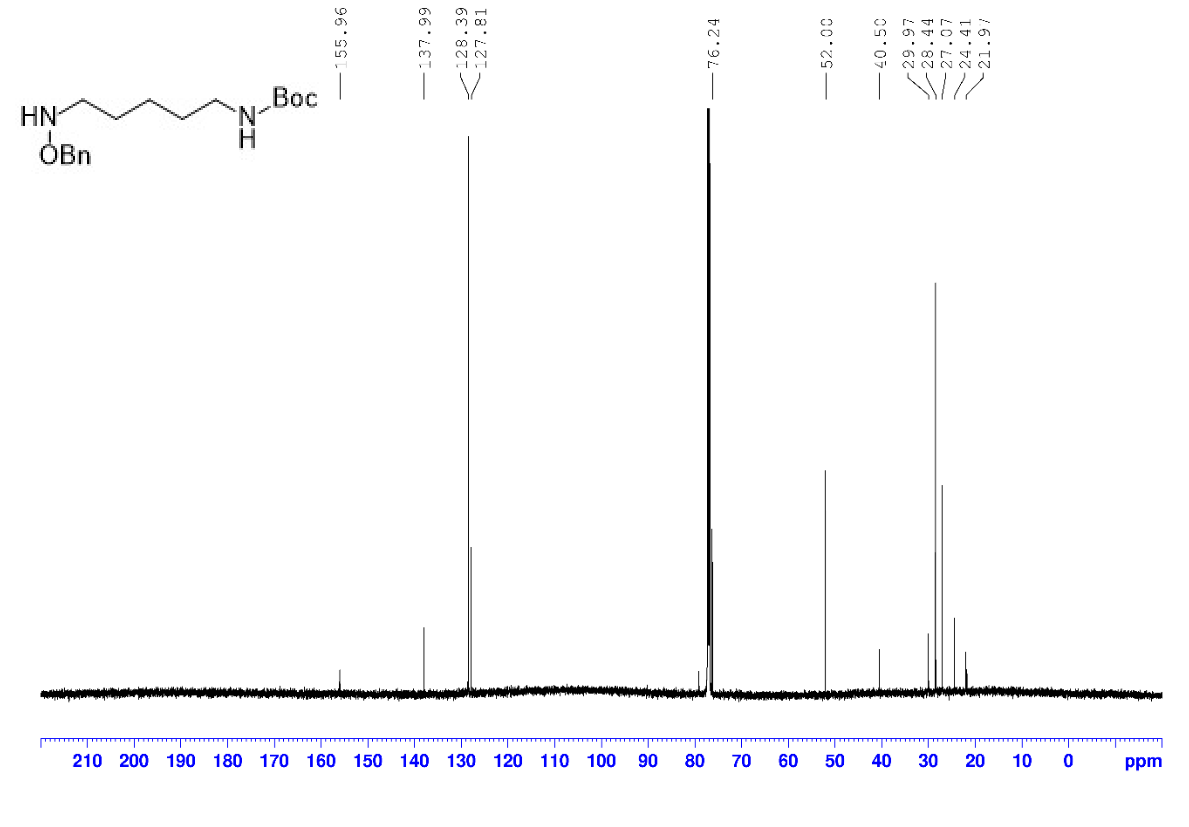

Figure S6. $^{13}C\{^{1}H\}$ NMR (150 MHz, $CDCl_3$) spectrum of **10**

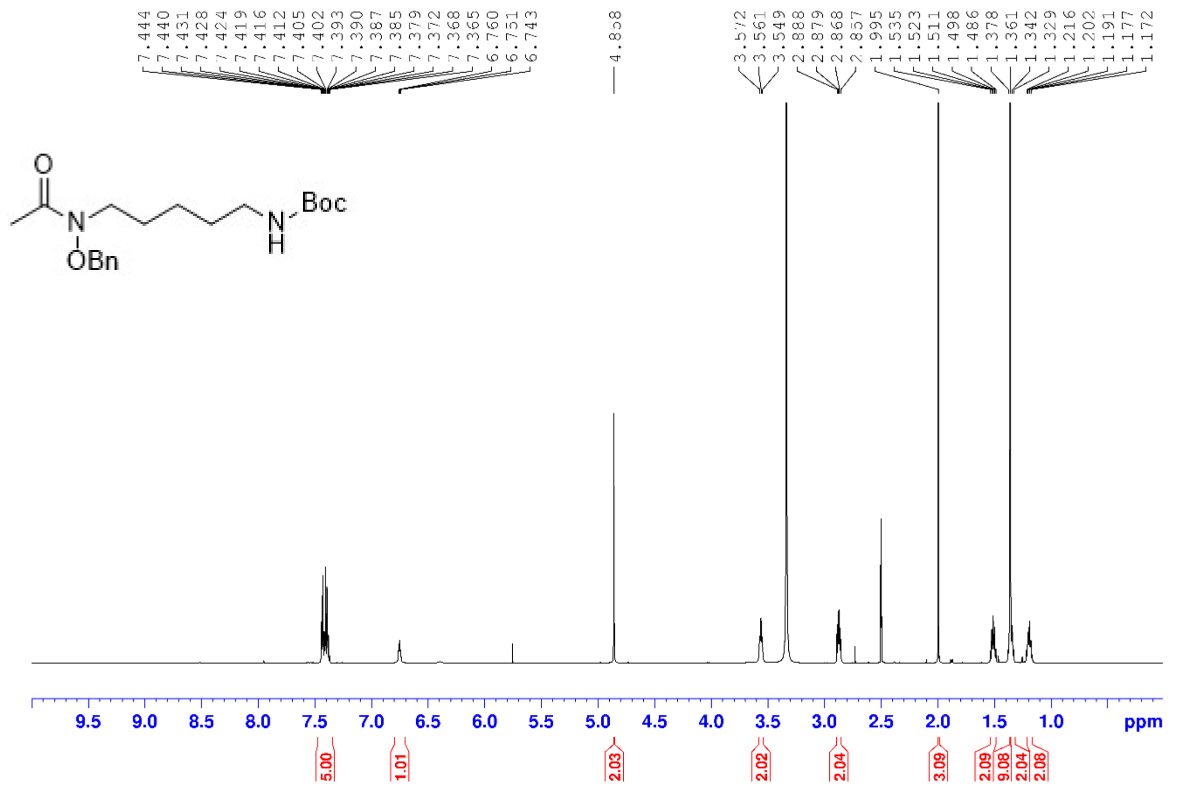

Figure S7. ^{1}H NMR (600 MHz, DMSO-d_6) spectrum of **11**

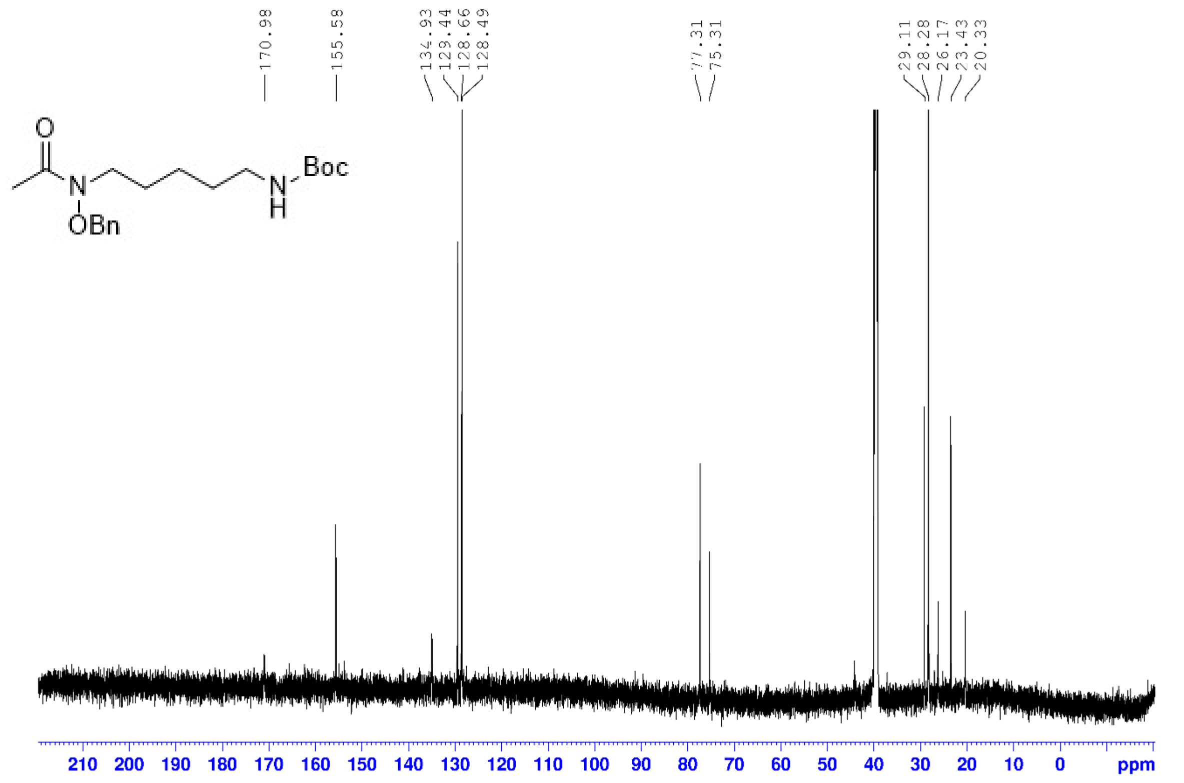

Figure S8. $^{13}C\{^{1}H\}$ NMR (150 MHz, DMSO-d_6) spectrum of **11**

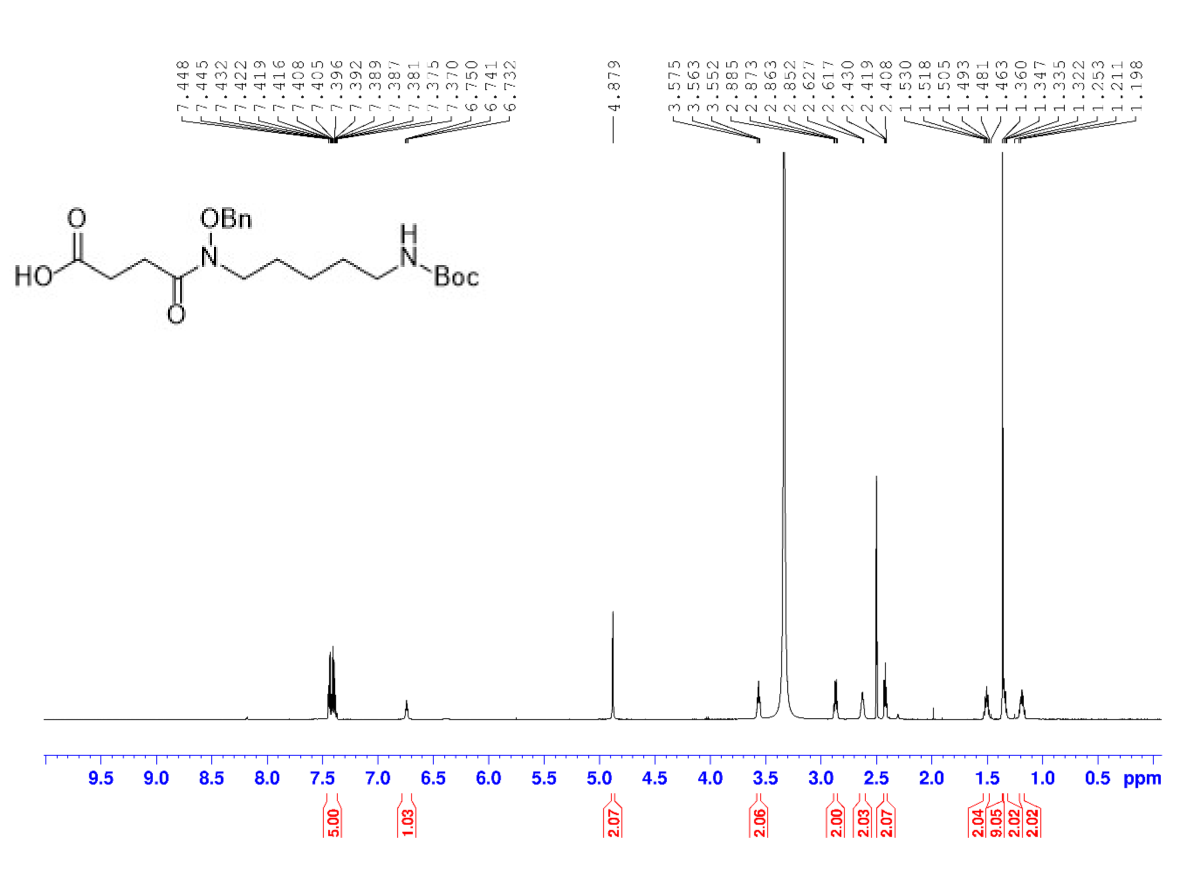

Figure S9. ^{1}H NMR (600 MHz, DMSO-d_6) spectrum of **12**

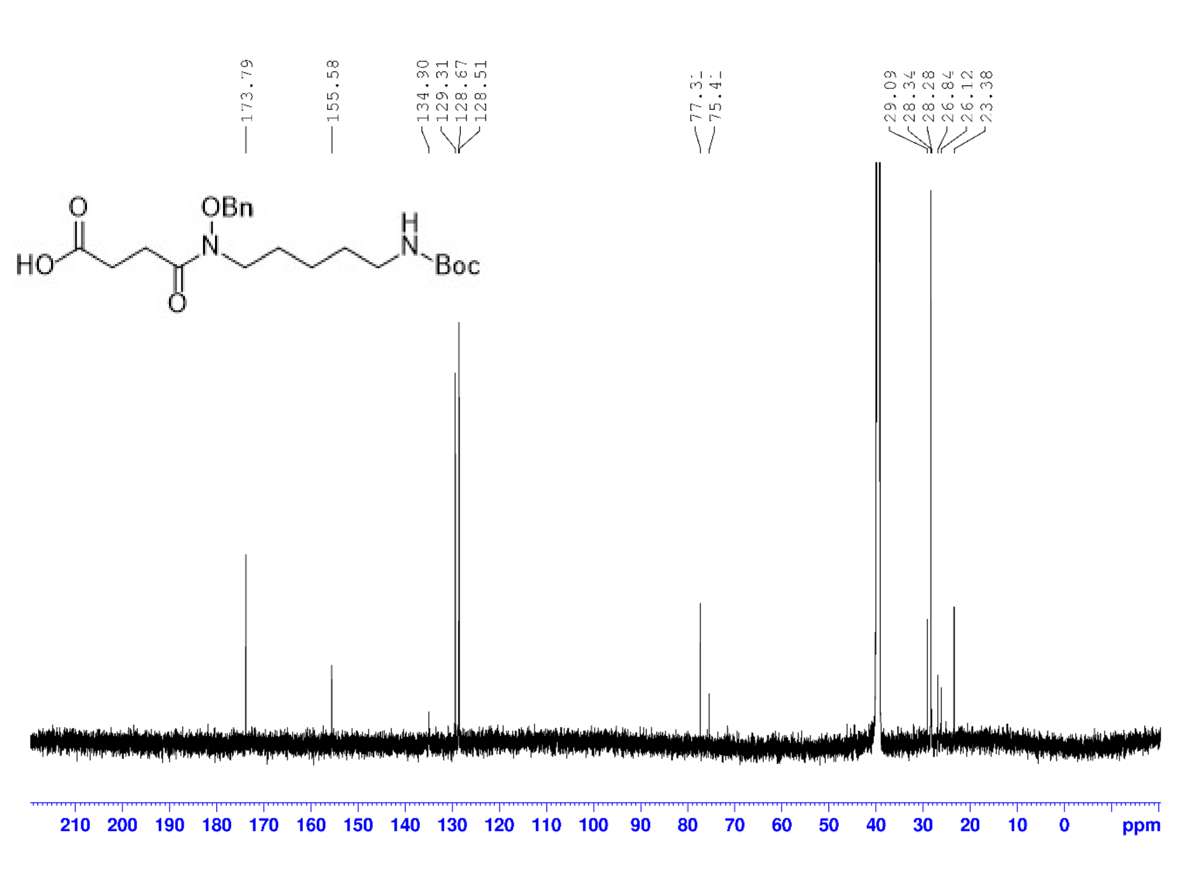

Figure S10. $^{13}C\{^{1}H\}$ NMR (150 MHz, DMSO-d_6) spectrum of **12**

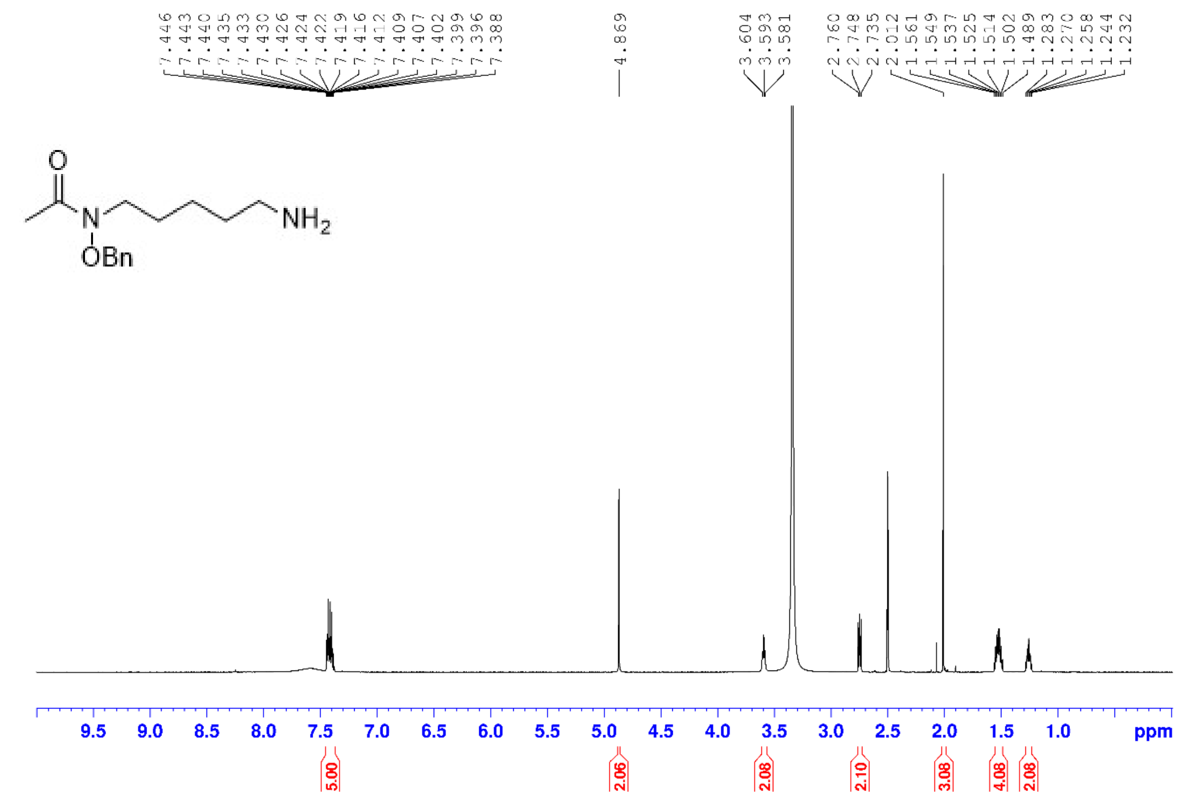

Figure S11. ^{1}H NMR (600 MHz, DMSO-d$_6$) spectrum of **13**

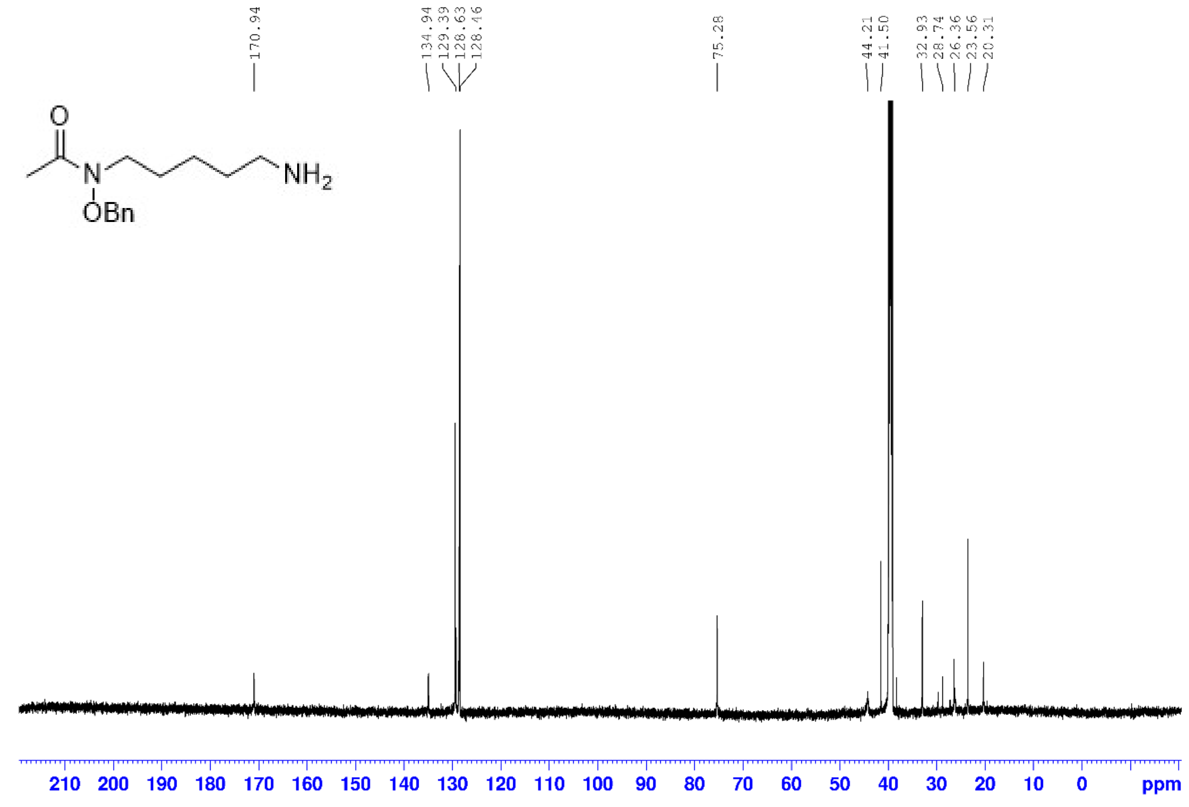

Figure S12. ^{13}C{^{1}H} NMR (150 MHz, DMSO-d$_6$) spectrum of **13**

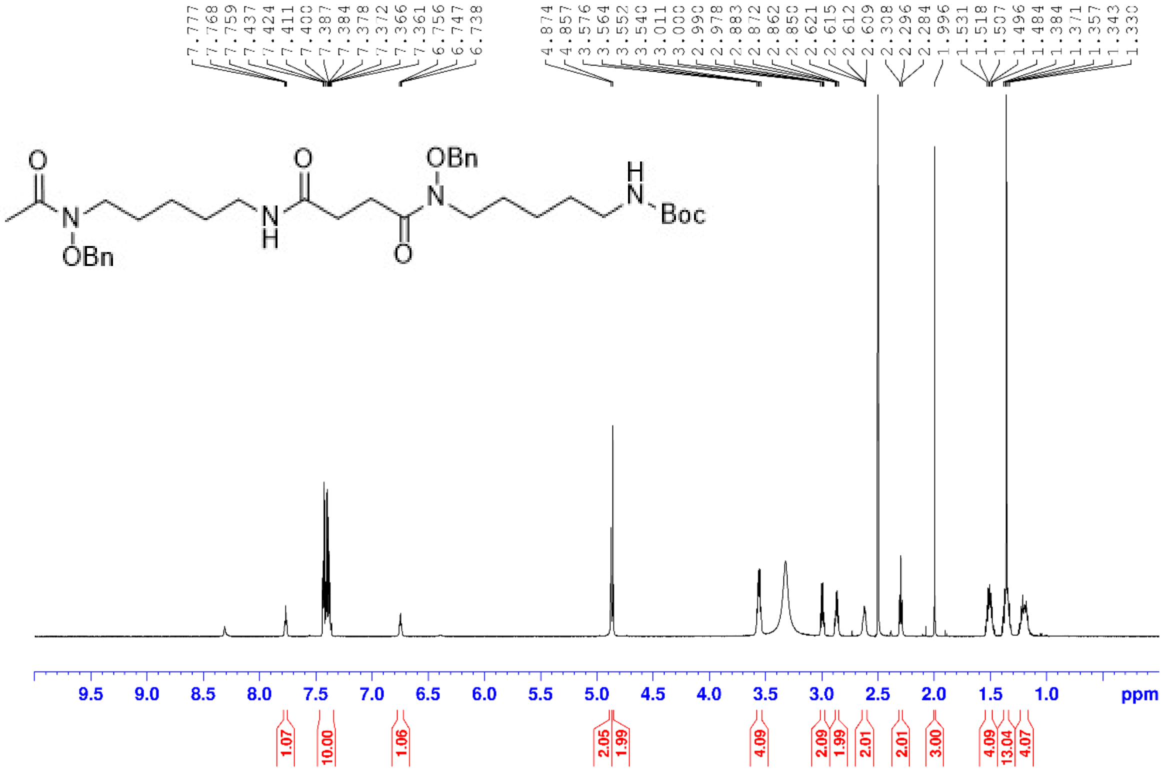

Figure S13. ^{1}H NMR (600 MHz, DMSO-d$_6$) spectrum of **14**

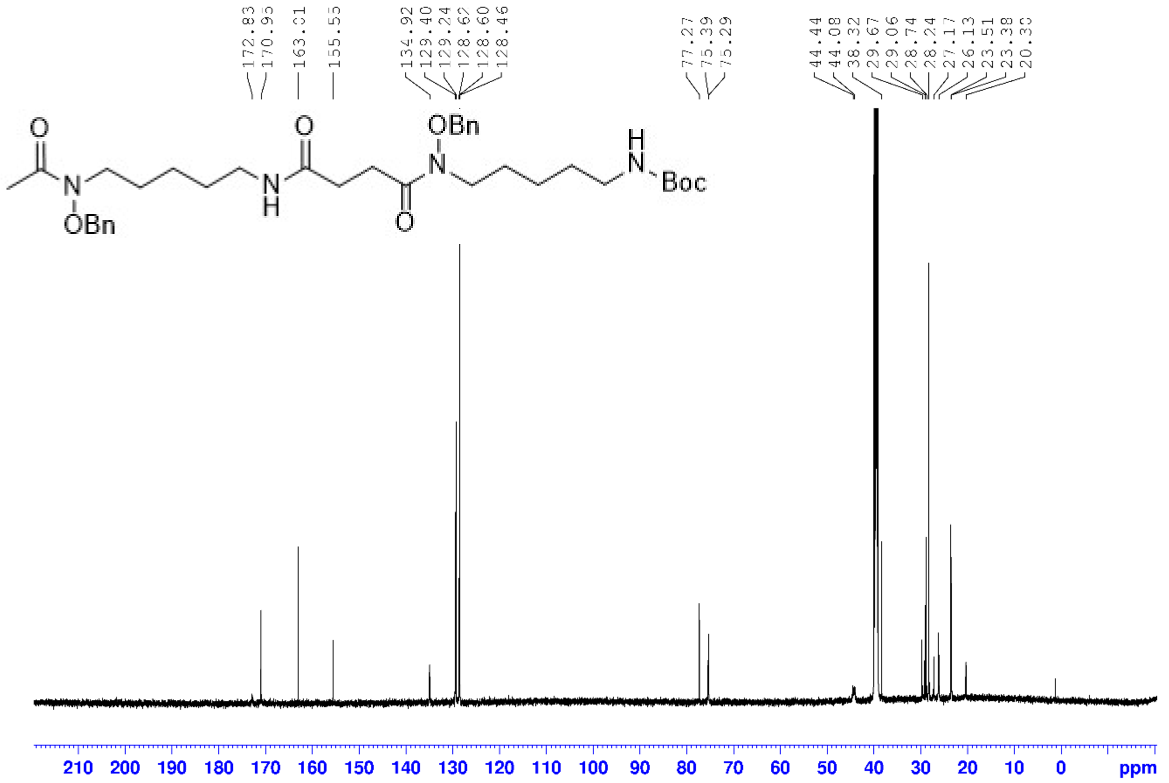

Figure S14. ^{13}C{^{1}H} NMR (150 MHz, DMSO-d$_6$) spectrum of **14**

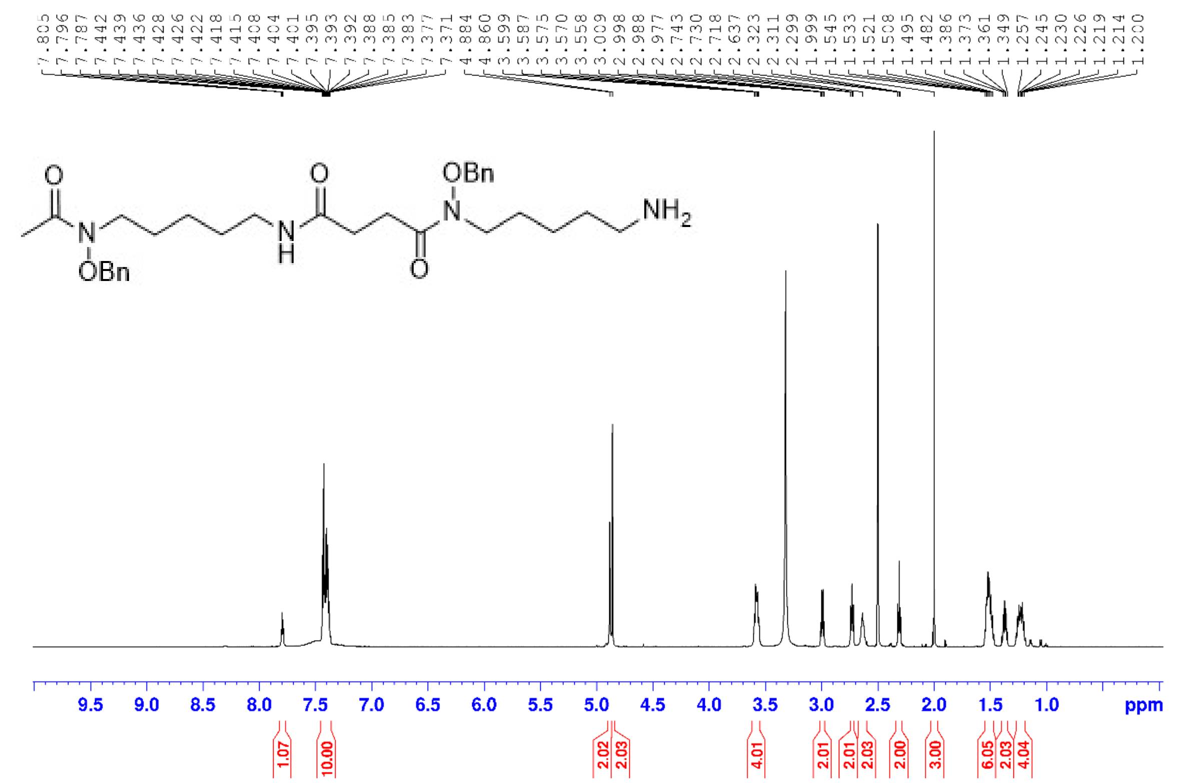

Figure S15. ^{1}H NMR (600 MHz, DMSO-d$_6$) spectrum of **15**

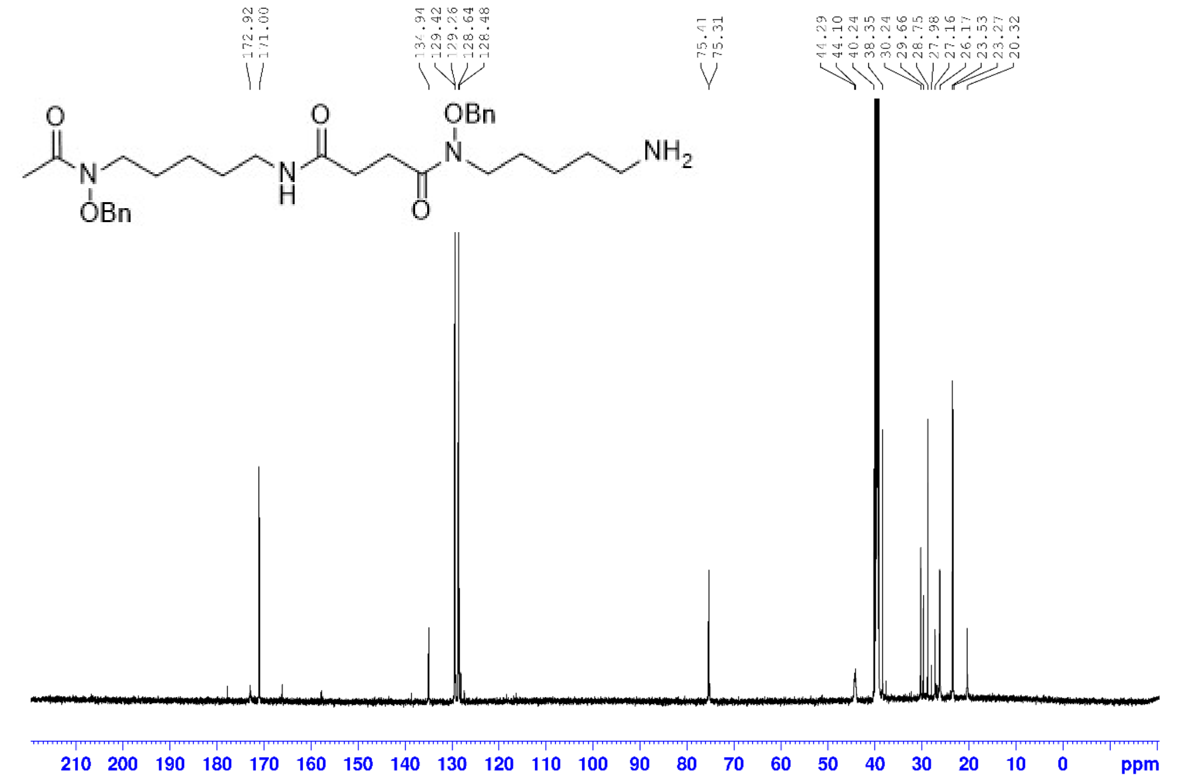

Figure S16. ^{13}C{^{1}H} NMR (150 MHz, DMSO-d$_6$) spectrum of **15**

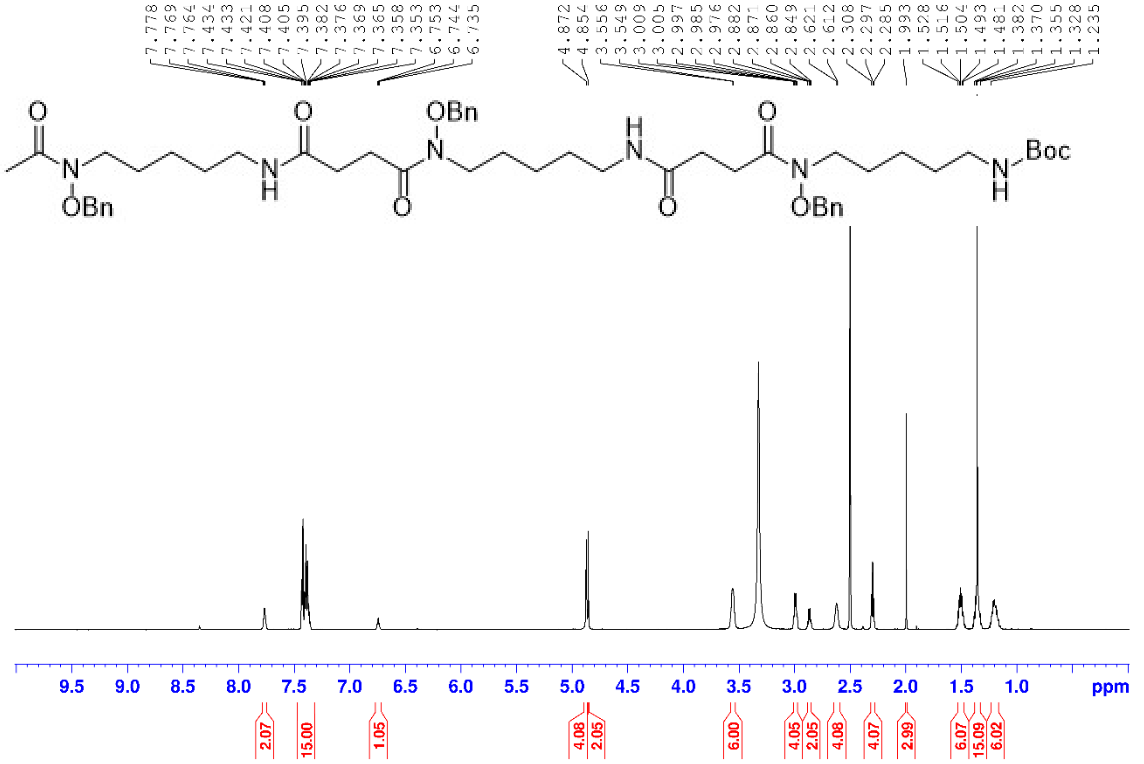

Figure S17. ^{1}H NMR (600 MHz, DMSO-d$_6$) spectrum of **16**

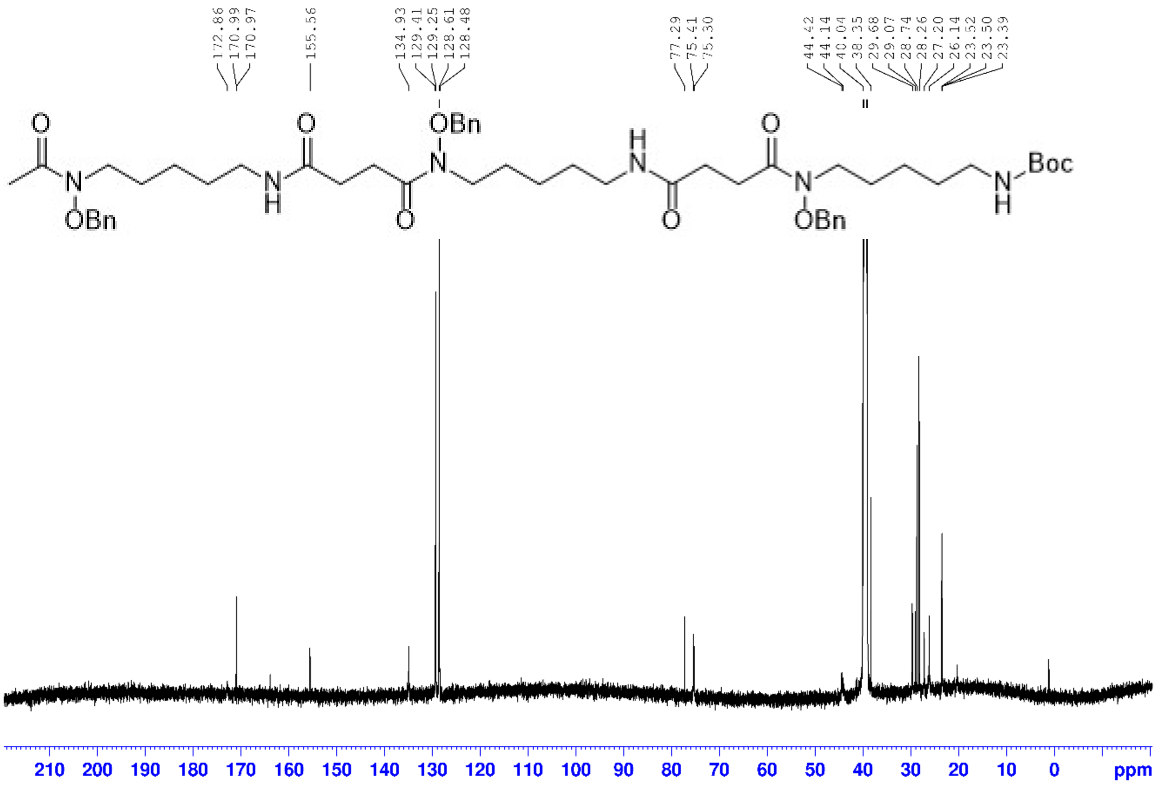

Figure S18. ^{13}C{^{1}H} NMR (150 MHz, DMSO-d$_6$) spectrum of **16**

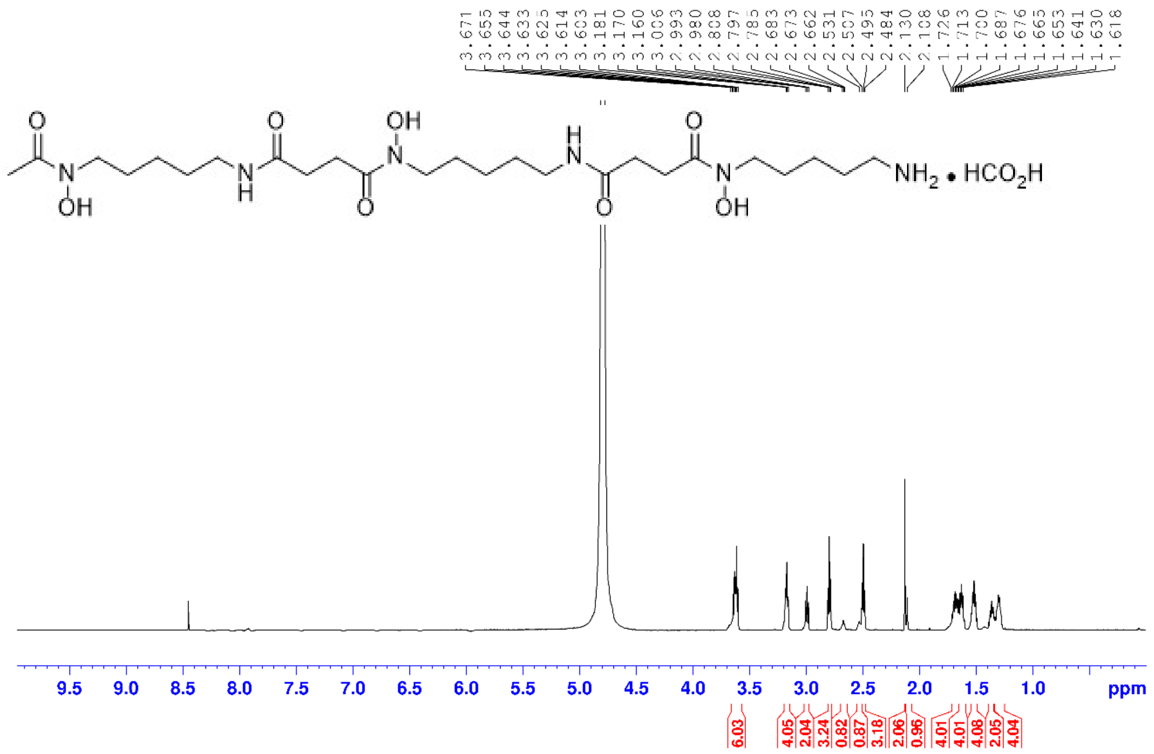

Figure S19. ^{1}H NMR (600 MHz, D_2O) spectrum of **1** formate salt

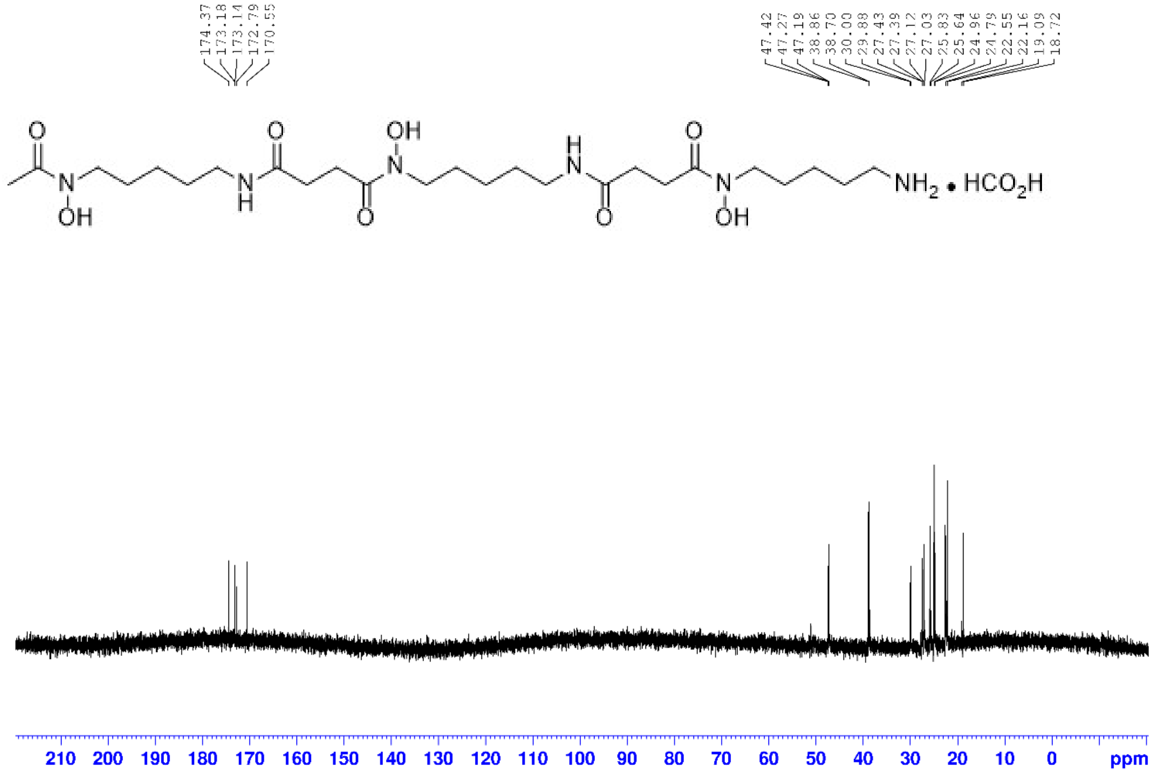

Figure S20. $^{13}C\{^{1}H\}$ NMR (150 MHz, D_2O) spectrum of **1** formate salt
